# The CENP-A chaperone complex spatially organizes centromeres

**DOI:** 10.1126/sciadv.aef1579

**Published:** 2026-09-09

**Authors:** Hindol Gupta, Julian Haase, Yicong Wu, Jiji Chen, Warif El Yakoubi, George Karadimov, Ana Losada, Takashi Akera, Hari Shroff, Alexander E. Kelly

**Affiliations:** ^1^ Laboratory of Biochemistry & Molecular Biology, Center for Cancer Research, National Cancer Institute, NIH, Bethesda, MD 20892, USA.; ^2^ Advanced Imaging and Microscopy Resource, National Institute of Biomedical Imaging and Bioengineering, NIH, Bethesda, MD 20892, USA.; ^3^ Cell and Developmental Biology Center, National Heart, Lung, and Blood Institute, National Institutes of Health, Bethesda, MD 20894, USA.; ^4^ Chromosome Dynamics Group, Molecular Oncology Programme, Spanish National Cancer Research Centre (CNIO), Madrid 28029, Spain.; ^5^ Janelia Research Campus, HHMI, Ashburn, VA 20147, USA.

## Abstract

Centromeres, defined by CENP-A–containing nucleosomes, direct kinetochore assembly for spindle attachment. In mitosis, CENP-A and the constitutive centromere-associated network (CCAN) of the inner kinetochore are arranged into bipartite subdomains within clearings of chromatin. However, less is known about their architecture during interphase. We report here an unrecognized structural role for the CENP-A chaperone machinery in establishing interphase centromere architecture. In interphase, CENP-A and the CCAN assemble conserved shell-like structures that enclose a chromatin-poor central cavity. This cavity is occupied by the interphase-specific CENP-A chaperone complex, which promotes CENP-A assembly once per cell cycle. The presence of the chaperone complex, but not its CENP-A–incorporating activity, is required to generate both the shell architecture and chromatin clearing. The CCAN scaffold CENP-C exhibits radial organization throughout the structure and is essential for its formation, primarily by recruiting the HJURP chaperone. Our findings broaden the role of the CENP-A chaperone machinery to include the structural organization of interphase vertebrate centromeres, independent of CENP-A deposition.

## INTRODUCTION

Centromeres are specialized chromosomal elements that direct assembly of the kinetochore, which mediates spindle attachment to ensure accurate chromosome segregation (*1*). Centromeres are specified by nucleosomes containing the histone H3 variant CENP-A, which recruits the constitutive centromere-associated network (CCAN) proteins to build the inner kinetochore (*2*). At mitosis, the CCAN recruits the outer kinetochore, which directly interacts with spindle microtubules. Centromeres are epigenetically maintained by the CENP-A chaperone complex consisting of HJURP and its cofactors, Mis18α, Mis18β, and Mis18BP1 (*3*). In most eukaryotes, this chaperone machinery assembles new CENP-A nucleosomes during interphase to restore CENP-A levels after dilution by DNA replication in the previous cell cycle. The CCAN component CENP-C directs the CENP-A chaperone complex to sites containing preassembled CENP-A nucleosomes (*4*–*8*), a process regulated by kinases Polo-like kinase 1 (PLK1) and cyclin-dependent kinase 1 (CDK1) (*5*, *9*–*15*). Thus, the CCAN is important for CENP-A assembly in interphase and for the assembly of the complete kinetochore in mitosis.

In the core centromere, CENP-A nucleosomes are interspersed with canonical histone H3 nucleosomes (*16*), with only ∼100 CENP-A nucleosomes per chromatid on mitotic chromosomes in human cells (*17*). Despite this low density, CENP-A nucleosomes are closely organized in three dimensions and occupy the surface of mitotic chromosomes, which promotes the proper capture and attachment of spindle microtubules (*18*). How this three-dimensional (3D) arrangement is established is a major question in chromosome biology. A recent work has revealed that individual centromeres and kinetochores are partitioned into two subdomains by condensin and cohesin (*19*). Furthermore, cryo–electron tomography (cryo-ET) reconstructions of intact human mitotic centromeres revealed that each subdomain resides within a chromatin-clearing region, distinct from bulk chromatin (*20*). These clearings consist of ∼250 nucleosomes, of which about 50, presumably containing CENP-A, are directly engaged with the CCAN. CENP-C is required to maintain these clearings, which are thought to concentrate CCAN complexes to promote kinetochore formation and microtubule attachment. However, it remains unclear how these clearings arise and when they form. Super-resolution imaging in human cells has visualized a G_1_-specific rosette-like arrangement of CENP-A immediately following HJURP-mediated deposition (*21*), suggesting that a specific centromere arrangement exists before mitosis.

In this study, we examine the de novo assembly of centromere and kinetochore architecture in *Xenopus* embryo extracts using advanced super-resolution imaging and deep learning resolution enhancement. We find that CENP-A and CCAN subunits assemble ∼200- to 300-nm shell-like structures in interphase that enclose a chromatin-poor central cavity, which is occupied by the CENP-A chaperone complex. Super-resolution imaging of mouse granulosa cells confirms the existence of these structures in mammals. Chaperone presence, but not CENP-A incorporation, is required to generate both the shells and the chromatin clearing, and the CCAN scaffold CENP-C is radially oriented to connect CENP-A chromatin to the chaperone core and is essential for structure formation. Our results reveal a previously unrecognized structural role for the CENP-A chaperone machinery in establishing interphase centromere architecture.

## RESULTS

### The centromere and inner kinetochore form conserved shell-like structures during interphase

**Fig. 1.**
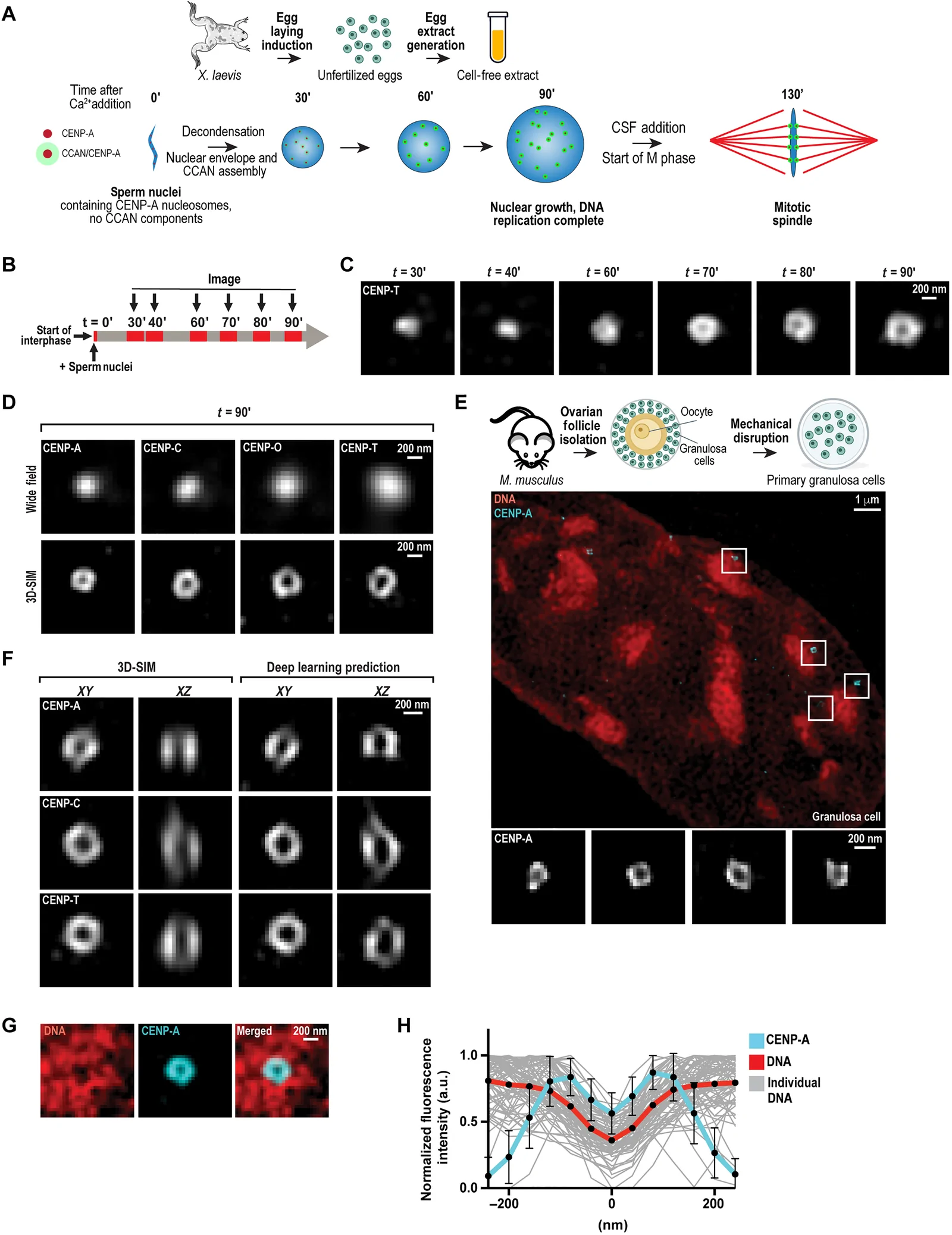
The centromere and inner kinetochore form shell-like structures around chromatin clearings in interphase. (**A**) Top: Schematic for preparing metaphase-arrested CSF extract from *X. laevis* eggs. Bottom: Schematic of the de novo CCAN assembly and cell cycle progression over the indicated timeline (in minutes) after adding *Xenopus* sperm nuclei and Ca^2+^. (**B**) Timeline for imaging CENP-T in interphase nuclei assembled in *Xenopus* egg extract. Samples were collected at the indicated times (minutes) after adding sperm and Ca^2+^ and processed for immunofluorescence. (**C**) Representative 3D-SIM immunofluorescence images of CENP-T at the indicated interphase time points. Scale bar, 200 nm. (**D**) Top: SIM-derived wide-field-equivalent images of CENP-A–, CENP-C–, CENP-O–, and CENP-T–labeled centromeres in interphase nuclei (*t* = 90′), generated from the raw SIM data by Fourier filtering to suppress SIM/moiré components. Bottom: Corresponding 3D-SIM reconstructions at the same magnification. (**E**) Top: Schematic for isolating mouse primary granulosa cells from *M. musculus.* Bottom: Representative 3D-SIM image of CENP-A (cyan) and DNA (DAPI; red) in nuclei from asynchronous mouse primary granulosa cells; boxed regions are magnified below (CENP-A; gray). (**F**) Lateral and axial views of representative 3D-SIM images of CENP-A, CENP-C, and CENP-T in interphase nuclei (*t* = 90′), before and after axial resolution enhancement by deep learning analysis. (**G**) Representative 3D-SIM image of DNA (DAPI; red) and CENP-A (cyan) in interphase nuclei (*t* = 90′), showing individual and merged fluorescence channels. (**H**) Line scans of DNA and CENP-A at centromeres from (G). Bold lines: mean intensity; thin gray lines: individual DNA traces, each aligned to the central minimum and normalized to its maximum. *n* = 75 centromeres (six nuclei). Representative data from one of two biological replicates shown throughout, unless otherwise noted. Scale bars, 200 nm and 1 μm [whole-nucleus view in (E)]. Error bars: SD. a.u., arbitrary units.

In mitosis, the CCAN and its associated CENP-A nucleosomes have a defined 3D arrangement within clearings of chromatin that is thought to promote proper kinetochore-microtubule attachment (*19*, *20*). However, less is known about how the CCAN and CENP-A are arranged during interphase. To explore this, we imaged the CCAN protein CENP-T throughout interphase by three-dimensional structured illumination microscopy (3D-SIM) in *Xenopus* egg extracts, which recapitulate fundamental biological processes and are amenable to strict cell cycle control with drug-free synchronization (Fig. 1A). We added sperm nuclei containing CENP-A nucleosomes but lacking kinetochore proteins (*22*) to cytostatic factor (CSF)–arrested egg extracts, added calcium to trigger release into interphase, and imaged CENP-T over 90 min, at which point replication is complete (Fig. 1B). As expected, no CENP-T signal was detected on sperm nuclei (fig. S1A). However, after 30 min, to allow for chromatin decondensation, nuclear assembly, and CCAN assembly, CENP-T was present at kinetochores (Fig. 1C and fig. S1A). CENP-T formed puncta in early interphase that expanded to ringlike structures by 60 min after the addition of calcium, reaching maximal size by 90 min (Fig. 1C). After release into M phase by addition of the fresh CSF-arrested extract, CENP-T rings were smaller but still observable after 10 min, a time analogous to prophase in somatic cells (fig. S1, B to D). However, they were no longer resolvable as rings at metaphase, when sister chromatids are fully separated (fig. S1C). CENP-C also transitioned from puncta to rings during interphase (fig. S1E). Rings were consistently observed at nearly all kinetochores (fig. S1F) and were detectable using different super-resolution technologies (fig. S1G). The formation of CENP-C and CENP-T rings in interphase did not depend on replication (fig. S1, H to K). Thus, the fundamental architecture of individual inner kinetochores in interphase is ringlike and is modulated throughout the cell cycle, independent of DNA replication.

We found that CENP-A and the CCAN protein CENP-O also formed stereotypical ringlike structures in interphase (Fig. 1D). Measures of the average ring diameter of CENP-A and CCAN structures revealed a hierarchy of nested rings wherein CENP-A and CENP-C rings had the smallest average diameter (204 ± 38 and 231 ± 34 nm, respectively), and CENP-O and CENP-T had the largest (257 ± 44 and 282 ± 38 nm, respectively) (fig. S2A). This suggests that the CCAN is assembled around CENP-A rings, with the spatial arrangement of CCAN subunits relative to CENP-A in agreement with cryo–electron microscopy (cryo-EM) studies on single CCAN–CENP-A–nucleosome complexes (*23*, *24*). Super-resolution imaging of an asynchronous population of mouse granulosa cells [Fig. 1E; ∼60 to 70% G_0_-G_1_ (*25*)] revealed that CENP-A forms ringlike structures during interphase. Thus, the ringlike arrangement of centromeric chromatin during interphase is a conserved principle in vertebrates, including mammals.

Although our 3D-SIM imaging of kinetochores from interphase *Xenopus* nuclei achieved lateral resolutions of ∼125 nm, the axial resolution was about three times poorer (∼340 to 380 nm). Therefore, we used a deep learning approach that yields isotropic 120-nm resolution on our 3D-SIM images of CENP-A and the CCAN (*26*). This approach predicts that CENP-A, CENP-C, and CENP-T form ringlike structures in both lateral and axial slices at the centroid of each structure (Fig. 1F). However, the arrangement of the signals in the axial dimension is less regular and slightly oblong when compared to the lateral slices. These data indicate that CENP-A and the CCAN form hollow, semienclosed particles in interphase.

Although CENP-A–containing chromatin is not present within these structures, canonical histone H3-containing chromatin might be. To test this, we coimaged DNA or histone H3 and CENP-A using 3D-SIM (Fig. 1, G and H, and fig. S2, B and C). The DNA levels were substantially reduced within the central cavity of the CENP-A structures (Fig. 1H), with chromatin density beginning to decrease at the outer diameter of the CENP-A ringlike structure. In agreement, histone H3 was also severely reduced within the central cavity surrounded by CENP-A chromatin (fig. S2, B and C). To further examine the relative location of DNA and CENP-A, we processed the 3D-SIM images using the deep learning protocol (fig. S2D). In resolution-enhanced images, there is an apparent decrease in DNA staining within the CENP-A shell and the central cavity. These data suggest a large chromatin clearing, with CENP-A shell-like structures defining its borders in all three dimensions.

### PLK1 activity is required for centromere architecture

**Fig. 2.**
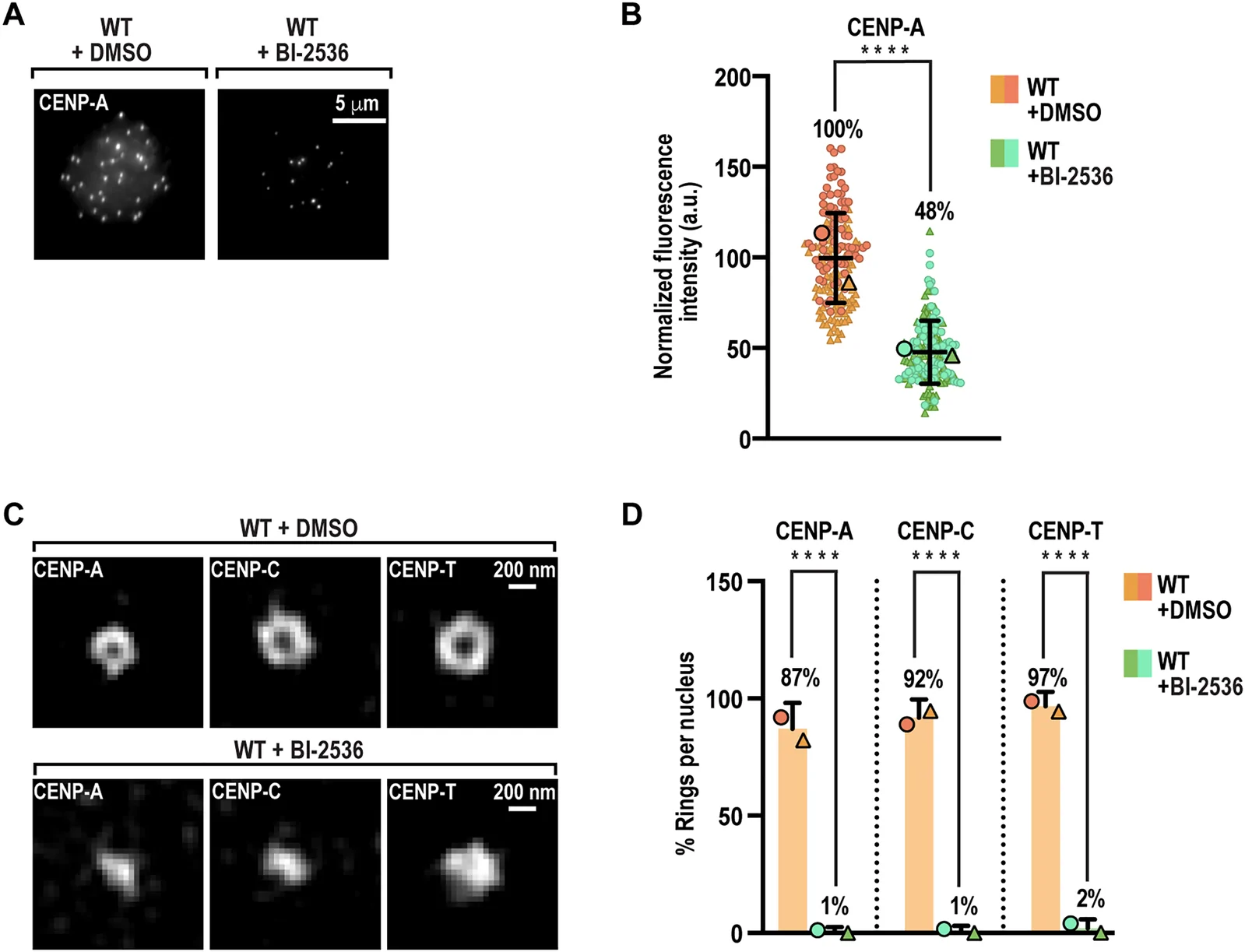
PLK1 inhibition perturbs centromere and kinetochore architecture. (**A**) Representative immunofluorescence images of CENP-A in interphase nuclei (*t* = 90′) from extract treated with DMSO or BI-2536. (**B**) CENP-A fluorescence intensity from (A), normalized to DMSO. *n* = 75 centromeres per condition (≥6 nuclei). (**C**) Representative 3D-SIM images of CENP-A, CENP-C, and CENP-T in interphase nuclei (*t* = 90′) from extract treated with DMSO or BI-2536. (**D**) Percentage of observable ringlike structures per nucleus for (C). *n* = 10 nuclei per condition (*n* ≥ 145 centromeres or kinetochores). Scale bars, 5 μm (A) and 200 nm (C). Graphs are SuperPlots (two biological replicates; means are color coded). Error bars: SD. a.u., arbitrary units. ****P < 0.0001.

During interphase in vertebrate cells, new CENP-A molecules are assembled at centromeres by the CENP-A chaperone complex to preserve centromere identity (*3*). The chaperone HJURP and the Mis18 complex (Mis18α, Mis18β, and Mis18BP1) constitute the chaperone complex that promotes the assembly of CENP-A into centromeric chromatin. Previous studies have demonstrated that PLK1 activity facilitates CENP-A assembly during interphase in human cells by promoting the complete assembly of the CENP-A chaperone complex at the centromere (*9*–*11*). Therefore, we tested whether PLK1 is essential for centromere architecture. In interphase egg extracts, inhibition of PLK1 with BI-2536 after exit from M phase decreased the total levels of CENP-A by nearly 50% (Fig. 2, A and B), similar to results observed in human cell culture (*11*). PLK1 inhibition also blocked the assembly of new CENP-A (fig. S3, A and B). Furthermore, BI-2536 treatment blocked the localization of HJURP and Mis18α to centromeres (fig. S3, C to F), highlighting the conservation of PLK1-dependent CENP-A assembly in the *Xenopus* egg extract system. Notably, PLK1 inhibition completely abrogated the formation of CENP-A, CENP-C, and CENP-T rings (Fig. 2, C and D) without affecting CENP-T or CENP-C levels at centromeres (fig. S3, G and H). This agrees with previous findings that a full complement of CENP-A is not required for CCAN assembly (*17*, *27*, *28*). Furthermore, the addition of BI-2536 had no effect on nuclear growth kinetics nor maximum nuclear area (fig. S4, A to D), indicating that the loss of centromere organization after PLK1 inhibition is not due to a delay or arrest in interphase progression. Thus, the PLK1-dependent control of the CENP-A chaperone complex is most likely necessary for the spatial arrangement of the centromere and the CCAN.

### HJURP controls centromere architecture independently of its role in CENP-A assembly

**Fig. 3.**
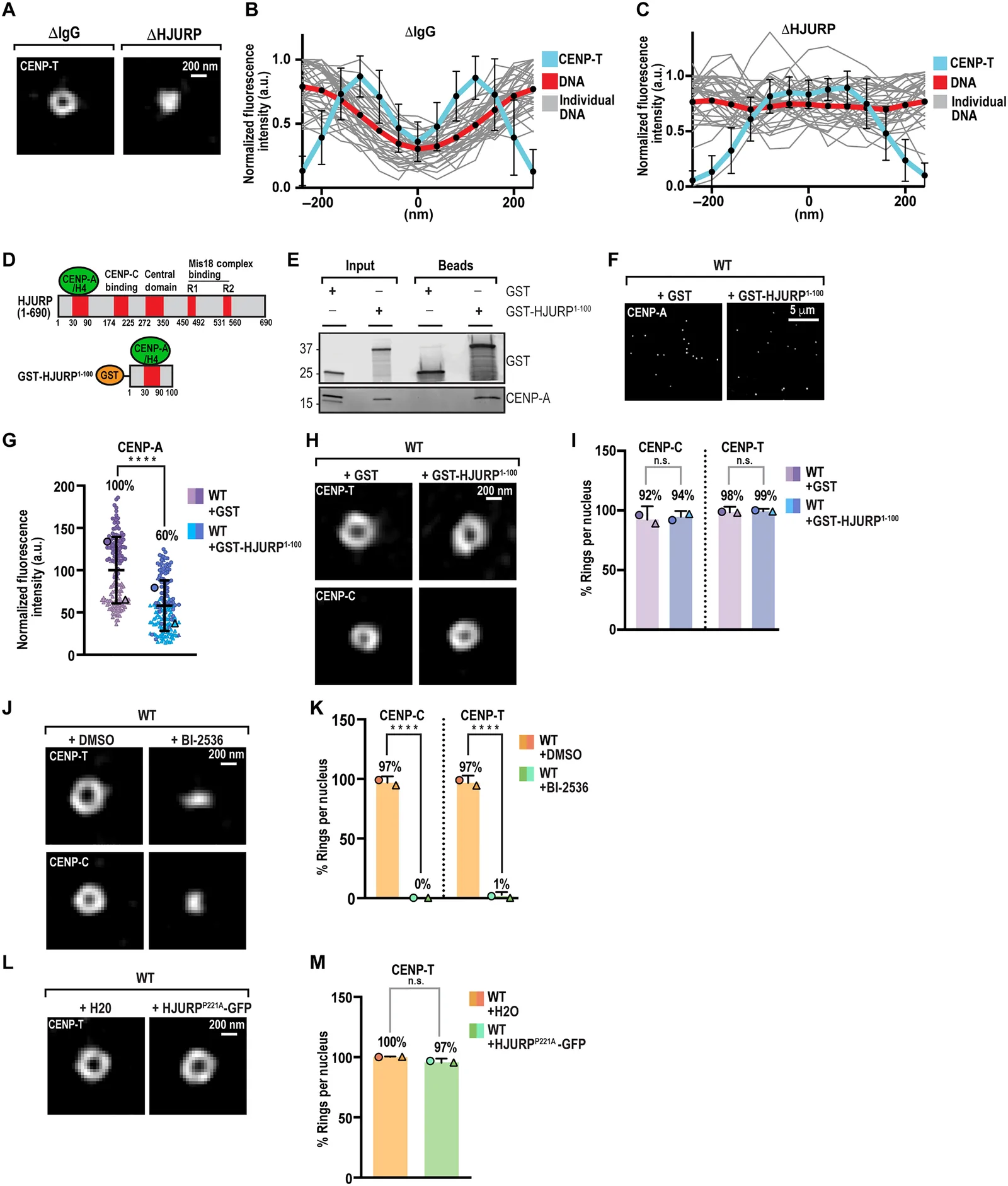
HJURP is required for centromere organization independently of CENP-A assembly. (**A**) Representative 3D-SIM images of CENP-T in interphase nuclei (*t* = 90′) from mock-depleted (ΔIgG) or HJURP-depleted (ΔHJURP) extracts. (**B** and **C**) Line scans of CENP-T and DNA at kinetochores in (A), aligned to the central minimum (ΔIgG) or maximum (ΔHJURP) of mean CENP-T intensity, each normalized to its maximum. *n* = 35 kinetochores per condition (four nuclei). (**D**) Top: HJURP domain organization: CENP-A/histone H4 dimer–, CENP-C–, and Mis18 complex–binding regions and the central domain. Bottom: GST-HJURP^1-100^ fragment, which binds CENP-A/H4. (**E**) Western blot for proteins that copurify with GST- or GST-HJURP^1-100^–coupled glutathione beads from HSS egg extracts lacking chromosomes. Input: 5%. (**F**) Representative immunofluorescence images of CENP-A in interphase nuclei (*t* = 90’) from extracts supplemented with purified GST or GST-HJURP^1-100^. Scale bar, 5 μm. (**G**) CENP-A fluorescence intensity from (F), normalized to GST. *n* = 75 centromeres per condition (≥6 nuclei). (**H**) Representative 3D-SIM images of CENP-C and CENP-T in interphase nuclei (*t* = 90′) from extract supplemented with GST or GST-HJURP^1-100^. (**I**) Percentage of observable ringlike structures per nucleus for conditions in (H). *n* = 10 nuclei per condition (≥145 centromeres). (**J**) Representative 3D-SIM images of CENP-C and CENP-T in interphase nuclei (*t* = 90′) from extract treated with DMSO or BI-2536. (**K**) Percentage of observable ringlike structures per nucleus for conditions in (J). *n* = 10 nuclei per condition (≥145 centromeres). (**L**) Representative 3D-SIM images of CENP-T in interphase nuclei (*t* = 90′), from extract supplemented with water (H_2_O) or HJURP^P221A^-GFP mRNA. (**M**) Percentage of observable ringlike structures per nucleus for conditions in (L). *n* = 10 nuclei per condition (≥145 centromeres). Scale bars, 200 nm and 5 μm (F). Graphs are SuperPlots (two biological replicates; means are color coded). Error bars: SD. A.u., arbitrary units. ****P < 0.0001; n.s., not significant.

PLK1 inhibition prevents CENP-A assembly by blocking the centromere localization of the Mis18 complex and HJURP during interphase (*9*–*11*). To directly test whether the chaperone complex is required for the spatial arrangement of centromeres, we immunodepleted HJURP from egg extracts (fig. S5A). HJURP depletion did not perturb interphase progression, as indicated by nuclear size at 90 min (fig. S5B). As expected (*7*, *29*), HJURP depletion prevented CENP-A assembly, as indicated by a nearly 50% decrease in total CENP-A at centromeres (fig. S5, C and D). HJURP depletion severely decreased the frequency of CENP-T ring formation in interphase, with CENP-T instead forming punctate structures (Fig. 3A and fig. S5E). Furthermore, the decrease in chromatin density in the central cavity of the centromere was lost upon HJURP depletion (Fig. 3, B and C, and fig. S5F). Thus, HJURP is required for the spatial arrangement of the centromere and for the clearing of chromatin at centromeres in interphase.

We asked whether the CENP-A assembly activity of HJURP is required for centromere and CCAN organization by perturbing HJURP’s ability to recruit CENP-A/histone H4 heterodimers, without affecting its localization to centromeres (*30*). To do this, we added an excess (*31*) of the CENP-A/histone H4-binding domain of HJURP fused to glutathione *S*-transferase (GST) (Fig. 3D; GST-HJURP^1-100^), which interacts with soluble CENP-A (Fig. 3E), to interphase extracts to sequester CENP-A/H4 heterodimers away from endogenous HJURP. This reduced total CENP-A at centromeres to levels similar to those observed after PLK1 inhibition (Fig. 3, F and G). However, unlike PLK1 inhibition, GST-HJURP^1-100^ did not affect the localization of Mis18α–green fluorescent protein (GFP) to centromeres (fig. S5, G and H). Thus, GST-HJURP^1-100^ blocks CENP-A assembly without perturbing the recruitment of chaperone complexes to centromeres. GST-HJURP^1-100^ did not affect the formation of CENP-C or CENP-T rings (Fig. 3, H and I), whereas PLK1 inhibition completely blocked ring formation (Fig. 3, J and K). To test whether CENP-A assembly can modulate the CCAN architecture, we sought to increase the amount of newly assembled CENP-A. A prior work has shown that the HJURP^P221A^ mutation increases new CENP-A assembly at centromeres (*32*). In agreement with these findings, expression of the hyperactive HJURP^P221A^ mutant in interphase extracts led to a 64% increase in CENP-A levels at centromeres over wild type (WT) (fig. S5, I and J). However, HJURP^P221A^-GFP did not affect the formation of CENP-T rings (Fig. 3, L and M). Together, our data suggest that the chaperone complex, but not CENP-A assembly, is required for the 3D organization of centromeres and the CCAN.

### A major pool of the CENP-A chaperone complex occupies the chromatin clearing

**Fig. 4.**
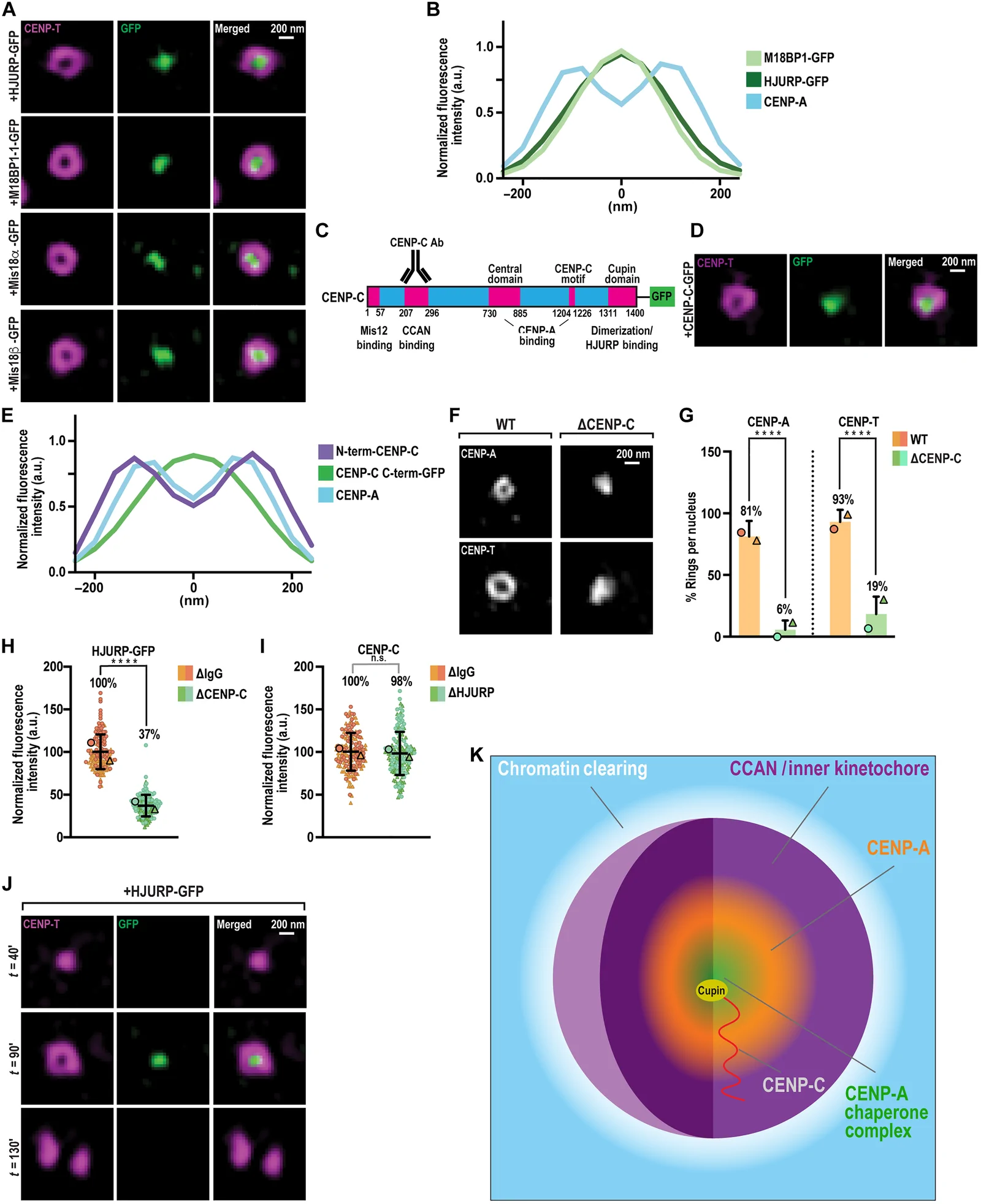
The CENP-A chaperone complex spatially organizes centromeric chromatin. (**A**) Representative 3D-SIM images of CENP-T (magenta) and GFP (green; GFP-Booster ATTO488) in interphase nuclei (*t* = 90′), assembled from extracts containing the indicated in vitro–translated GFP-tagged chaperone complex proteins. (**B**) Averaged, aligned line scans of M18BP1-GFP, HJURP-GFP, and CENP-A. (**C**) CENP-C domain organization: Mis12-binding, CCAN-binding, central domain, CENP-C motif, HJURP-binding, and dimerization domain; antibody (Ab) epitope (amino acids 207 to 296) indicated. (**D**) Representative 3D-SIM image of CENP-T (magenta) and GFP (green) in interphase nuclei, (*t* = 90′) from extracts containing in vitro–translated CENP-C–GFP. (**E**) Averaged, aligned line scans of CENP-C N-terminal epitope, CENP-C–GFP, and CENP-A. (**F**) Representative 3D-SIM images of CENP-A and CENP-T in interphase nuclei (*t* = 90′) assembled from WT or ΔCENP-C extract. (**G**) Percentage of observable ringlike structures per nucleus for conditions in (F). *n* = 10 nuclei per condition (≥145 centromeres). (**H**) HJURP-GFP fluorescence intensity in interphase nuclei (*t* = 90′), assembled from the ΔIgG or ΔCENP-C extract containing in vitro–translated HJURP-GFP, normalized to ΔIgG. *n* = 75 kinetochores per condition (≥6 nuclei). (**I**) CENP-C fluorescence intensity in interphase nuclei (*t* = 90′), assembled from the ΔIgG and ΔHJURP extract, normalized to ΔIgG. *n* = 75 kinetochores per condition (≥6 nuclei). (**J**) Representative 3D-SIM images of CENP-T (magenta) and HJURP-GFP (green) in interphase nuclei and mitotic chromosomes (*t* = 40′ and 90′, interphase; 130′, metaphase), assembled from WT extract containing in vitro–translated HJURP-GFP. (**K**) Model of interphase centromere/kinetochore organization. Concentric zones of CENP-A–enriched chromatin and the CCAN are arranged by the presence of the chaperone machinery in a chromatin-sparse cavity. CENP-C spans the structure, its C-terminal cupin domain within the cavity, and its N-terminal region within the CENP-A/CCAN shells. Scale bars, 200 nm. Graphs are SuperPlots (two biological replicates; means are color coded). Error bars: SD. a.u., arbitrary units. ****P < 0.0001; n.s., not significant.

Our data indicate that the chaperone complex promotes the spatial arrangement of centromeric chromatin, which creates a chromatin clearing within these structures. To further understand how the chaperone complex does this, we used 3D-SIM imaging combined with anti-GFP nanobody labeling to analyze the spatial distribution of GFP-tagged chaperone complex proteins relative to the CCAN protein CENP-T. Unlike CENP-A and the CCAN, which form continuous ringlike structures in the XY plane, each member of the chaperone complex localizes as discrete punctate structures within the interior of CENP-T rings (Fig. 4A) with a unimodal distribution of fluorescence intensity for both HJURP-GFP and Mis18BP1-GFP that peaks at the local minimum between the bimodal peaks of CENP-T, similar to the previously observed CENP-A “rosettes” surrounding HJURP signals in somatic cells (*21*) (fig. S6, A and B). CENP-T–GFP formed ringlike structures similar to those observed with antibodies targeting endogenous CENP-T and CENP-C, demonstrating the validity of anti-GFP nanobody labeling (Fig. 1D and fig. S6, C to E). Although HJURP-GFP and Mis18BP1-GFP signals were well separated from CENP-T, they partially overlapped with the smaller CENP-A ring structures (Fig. 4B), in agreement with current models of CENP-A assembly in which HJURP and the Mis18 complex localize to preexisting CENP-A nucleosomes to replace nearby histone H3.3 “placeholder” nucleosomes with new CENP-A molecules from the soluble pool (*13*, *33*–*37*). Thus, our data indicate that, although HJURP and the Mis18 complex engage with CENP-A chromatin, they primarily occupy the chromatin-free central cavity within CENP-A chromatin shells.

### CENP-C spans from the chromatin clearing to CENP-A chromatin and the CCAN

CENP-C interacts with preexisting CENP-A nucleosomes and is an important regulator of CENP-A assembly (*7*, *11*, *38*), suggesting it may contribute to centromere and kinetochore architecture during interphase. CENP-C promotes CENP-A assembly, in part, by recruiting HJURP to centromeres through a direct interaction between the CENP-C C-terminal cupin domain and HJURP (*32*, *39*, *40*). Given the direct interaction between CENP-C and HJURP, it was unexpected that HJURP exhibited a notably different localization pattern compared to CENP-C (Figs. 1D and 4A). CENP-C is a modular scaffold protein containing distinct, evolutionarily conserved domains that bind the outer kinetochore, the CCAN, CENP-A nucleosomes, and HJURP (*22*, *32*, *41*) (Fig. 4C). Our anti–CENP-C antibody targets an epitope within CENP-C that overlaps with the CENP-HIKM and CENP-NL binding regions (*22*, *42*). This epitope is ∼1000 residues away from its C-terminal cupin domain, which is responsible for HJURP binding (*32*), suggesting that the CENP-C C terminus may localize differently from the rest of the CCAN. To test this, we tagged the CENP-C C terminus with GFP immediately downstream of the cupin domain and determined its localization relative to the CCAN using an anti-GFP nanobody. Notably, the CENP-C C terminus localized within the center of CENP-T rings (Fig. 4D and fig. S6F), exhibiting a unimodal fluorescence distribution similar to that observed for HJURP-GFP and M18BP1-GFP (fig. S6G). Further analysis of the relative positions of the CENP-C N and C termini with respect to CENP-A indicates that the CENP-C C terminus not only primarily localizes to the central chromatin clearing but also overlaps with CENP-A (Fig. 4E). To confirm these findings, we depleted CENP-C, added back full-length CENP-C–GFP, and determined the localization of its C-terminal GFP tag relative to the N-terminal CENP-C epitope through simultaneous two-color imaging. The CENP-C C terminus localized within the center of CENP-C rings (fig. S6, H and I). Thus, CENP-C adopts an extended and likely radially oriented conformation, positioning its HJURP-binding C terminus primarily within the central cavity of the centromeric chromatin structure. At the same time, its CCAN-scaffolding N terminus forms a peripheral shell around CENP-A chromatin.

### CENP-C promotes centromere organization through recruitment of HJURP to centromeres

To test whether CENP-C is required for centromere architecture, we depleted CENP-C from egg extracts. As expected, CENP-C depletion prevented new CENP-A assembly, reducing total centromeric CENP-A levels by nearly 50% compared to controls (fig. S7, A to C). CENP-C depletion did not alter nuclear size, indicating that it did not perturb interphase progression (fig. S7D). Loss of CENP-C drastically decreased the frequency of CENP-A and CENP-T rings (Fig. 4, F and G). Instead, in the absence of CENP-C, CENP-A and CENP-T formed irregular puncta (Fig. 4F) that resemble their early interphase spatial arrangement (Fig. 1C). In contrast, depletion of CENP-T minimally affected the formation of CENP-A and CENP-C rings (fig. S7, E to I). These findings demonstrate that CCAN protein CENP-C specifically controls the higher-order, 3D organization of CENP-A and the CCAN during interphase.

To further understand the role of CENP-C in promoting centromere architecture, we measured HJURP-GFP levels at centromeres in mock-depleted and CENP-C–depleted egg extracts (Fig. 4H and fig. S7J). Consistent with a prior work (*7*), CENP-C depletion led to a 63% reduction in HJURP-GFP at centromeres, without affecting total HJURP protein levels (fig. S6I). Conversely, HJURP depletion did not perturb CENP-C levels at kinetochores (Fig. 4I and fig. S7K). Furthermore, although CENP-C is present at centromeres throughout the cell cycle, HJURP presence is strictly correlated with the shell-like structure of centromeres in interphase (Fig. 4J and fig. S7L). We could not detect HJURP at centromeres early in interphase, before the formation of observable rings (Fig. 1C), nor during metaphase, when rings have collapsed (fig. S1C). Together, these data show that HJURP is necessary for centromere architecture and suggest that the main role of CENP-C in the 3D arrangement of centromeric chromatin is through HJURP recruitment. Other functions of CENP-C, such as recruitment of the Mis18 complex or oligomerization, may also contribute to this process (*40*).

## DISCUSSION

Advances in assembling repetitive sequences have clarified where CENP-A resides along centromeric DNA (*43*–*45*), but how these 1D positions are organized into a 3D, functional centromere is less well understood (*46*). We reveal a structural role for the CENP-A chaperone complex that is independent of its canonical deposition function. In interphase, HJURP and the Mis18 complex occupy a chromatin-poor central cavity encircled by concentric zones of CENP-A nucleosomes and the CCAN. The CCAN protein CENP-C spans this assembly in a defined N-to-C orientation, linking the chaperone core to CENP-A chromatin and the broader CCAN, and is required for the formation of interphase centromere architecture, likely through the direct recruitment of HJURP (Fig. 4K). We show that these structures are present in both *Xenopus* egg extract nuclei and in primary mouse granulosa cells. CCAN shell-like structures have been reported in chicken DT40 cells (*47*), and CENP-A “rosettes” encircle HJURP in human cells (*21*). Therefore, the shell-like arrangement of CENP-A and the CCAN is conserved among vertebrates, and it is highly likely that the HJURP-mediated and the Mis18 complex–mediated organization of centromeres is also a conserved mechanism.

Our data indicate that a cell cycle–dependent mechanism regulates centromere architecture. PLK1-dependent chaperone assembly at centromeres during interphase creates a chromatin clearing encircled by CENP-A–enriched chromatin and the CCAN. Prior studies show that PLK1-driven assembly is antagonized by CDK, which evicts HJURP and other chaperone components via phosphorylation (*5*, *9*–*11*, *13*). In *Xenopus* egg extracts, chaperone eviction occurs upon mitotic entry, driven in part by CDK-dependent disruption of the HJURP–CENP-C interaction (*32*). Consistent with this, we observe that loss of the shell-like architecture in mitosis coincides with loss of HJURP from centromeres (Fig. 4J and fig. S7L), supporting a model in which CDK-dependent chaperone removal at mitosis results in the collapse of the extended CENP-A/CCAN shells into smaller puncta, which, in turn, serve as sites for outer kinetochore assembly. In somatic human cells, where CENP-A loading is confined to G_1_, CDK activity during S and G_2_ is sufficient to reverse PLK1-enabled recruitment (*12*). Super-resolution imaging in U2OS cells has revealed a rosette-like CENP-A structure surrounding HJURP in G_1_, which is lost upon G_1_ exit (*21*). Thus, PLK1-dependent formation of centromere shells and their CDK-dependent collapse are most likely general features of vertebrate centromeres.

The interphase organization of centromeric chromatin and the CCAN likely affects centromere and kinetochore function in mitosis. Studies in human cells have defined mitotic centromere architecture: One showed that individual centromeres comprise two subdomains of chromatin loops forming a bipartite structure stabilized by the SMC complexes condensin and cohesin (*19*); another identified chromatin clearings that demarcate CENP-A and inner kinetochore (CCAN) territories and demonstrated a requirement for CENP-C to maintain these clearings (*20*). A complementary work reported ringlike arrangements of α-satellite DNA and CENP-A at mitotic centromeres in human cells (*48*) and that outer kinetochore proteins form ringlike structures in mitotic *Xenopus* egg extracts (*49*). Against this backdrop, our results support a model in which the chaperone complex creates chromatin clearings surrounded by radially organized shells or zones of CENP-A and the CCAN in interphase, and these clearings function as “placeholders” into which CENP-A chromatin and the CCAN redistribute after chaperone eviction, generating the mitotic chromatin clearings and architectures described by others. This chaperone-dependent architecture formed in interphase may also template assembly of the outer kinetochore, thereby promoting proper attachment of kinetochores to spindle microtubules. We emphasize that our evidence establishes temporal correlation and architectural continuity but does not directly demonstrate that interphase clearings are the immediate precursors of mitotic clearings.

The interphase arrangement of CENP-A and the chaperone complex may also be necessary for efficient and accurate CENP-A assembly. A predominant model posits that a preexisting CENP-A nucleosome templates assembly of a new CENP-A nucleosome to restore prereplicative CENP-A levels; proximal histone H3.3 nucleosomes serve as “placeholders” that are evicted and replaced by HJURP-mediated delivery of CENP-A/H4 heterodimers (*13*, *33*–*37*). How this eviction and replacement are targeted and how centromere drift and expansion are prevented remain unclear (*46*). Although we demonstrate that CENP-A assembly is not required for centromere architecture, the shell-like architecture may regulate assembly in two ways. First, the high local concentration of chaperone complexes in a defined space near and colocalized with preexisting CENP-A nucleosomes may accelerate eviction and assembly. Second, sequestering the chaperone complex away from bulk chromatin could prevent CENP-A loading at noncentromeric sites and limit loading at centromeres. Although heterochromatin and other factors act as local boundaries to prevent centromere drift and expansion (*50*, *51*), current models emphasize 1D spreading along linear DNA. Our results raise the possibility that centromere drift and expansion are further delimited in three dimensions by the spatial organization of CENP-A chromatin and chaperone complexes, potentially creating a 3D zone of eviction and assembly.

How might the chaperone complex organize centromeric chromatin into shell-like structures around chromatin clearings in interphase, independent of new CENP-A assembly? Our findings suggest that the chaperone complex excludes bulk chromatin while maintaining the proximal organization of CENP-A–enriched chromatin. The cupin domain of CENP-C, which interacts with HJURP (*32*, *40*), resides within these structures and forms higher-order oligomers that promote self-association of centromeric chromatin (*52*). It is therefore possible that CENP-C oligomers cluster CENP-A nucleosomes and help to radially repel these clusters to form shells. However, our data demonstrate that HJURP is necessary for these structures to form and that CENP-C alone is insufficient to promote the 3D organization of centromeres in interphase. In total, our results suggest that HJURP, and possibly the Mis18 complex, dictate centromere organization in interphase. This does not rule out the possibility that CENP-C oligomerization may play an important role, beyond HJURP recruitment, in organizing chaperone complexes to effect centromere organization. Phase separation and electrostatic repulsion have been proposed to promote centromere organization through self-association of centromeric chromatin and repulsive forces generated by kinetochore proteins (*53*). However, the oriented N-to-C arrangement of CENP-C molecules we observe (Fig. 4, D and E, and fig. S6H) and the defined stoichiometry of the HJURP/Mis18 complex (*34*, *54*) argue against liquid-liquid phase separation, which relies on largely nonspecific, dynamic interactions, as the main organizing principle (*55*). Alternatively, the chaperone complex may form a structural foundation that organizes CENP-A chromatin. This model is analogous to centriole organization (*56*–*58*), in which proteins such as SAS-6 provide a defined architecture to organize centriolar microtubules. Thus, centromeres and kinetochores may share organizational principles with centrioles and centrosomes.

Several limitations of our study frame the work needed to extend it. For instance, we did not test PLK1 or chaperone dependence outside of the egg extract system, which will require perturbation and live-cell imaging in mammalian cells. Our description of a hollow shell surrounding a chromatin-poor cavity is inferred from the distributions of labeled proteins imaged by 3D-SIM and enhanced by deep learning rather than from nucleosome-resolution structural data, and approaches such as cryo-ET will be needed to define the geometry and the basis of chromatin exclusion at nanometer resolution. Last, our functional model remains correlative: Although CENP-A incorporation is dispensable for building the structure, directly establishing that this architecture preconfigures mitotic centromeres or spatially delimits CENP-A loading will require separation-of-function perturbations that disrupt the shells while preserving chaperone levels and CENP-A deposition, together with assays of mitotic organization, chromosome segregation fidelity, and the site of CENP-A assembly.

## MATERIALS AND METHODS

### Cloning and plasmid construction

*Xenopus laevis* M18BP1 (isoform M18BP1.S), Mis18α, and Mis18β genes were synthesized (Twist Bioscience) and inserted into a pCS2+ expression vector containing a LAP tag, a combination of 6xHis tag and enhanced GFP, at the C terminus. 3xMyc–CENP-A was synthesized and inserted into a pCS2+ expression vector (Twist Bioscience). CENP-T, HJURP, and CENP-C were polymerase chain reaction amplified and cloned into a pCS2+ vector containing a C-terminal LAP tag by Gibson assembly (NEB). Residues 1 to 100 of HJURP (HJURP^1-100^) were synthesized and inserted (Twist Bioscience) into the pGEX-6P-1 vector. HJURP mutation P221A was generated by Q5 site-directed mutagenesis (NEB) using the primer 5′-CAAAGTCTCTGCCATGAAATTTCGAAG-3′.

### Generation of anti-xCENP-A serum for Western blotting

To generate serum against xCENP-A for Western Blotting, a peptide spanning residues 1 to 20 of *X. laevis* CENP-A including an additional cysteine residue for conjugation was synthesized, MRPGSTPPSRRKSRPPRRVSC-amide (Biosynth, MA, USA), and used to immunize rabbits (Labcorp Early Development Laboratories Inc., WI, USA).

### Xenopus laevis

WT *X. laevis* 9+-cm mature females and 7.5- to 9-cm mature males were obtained from Xenopus 1 (Michigan, USA). The animals were maintained and handled according to the approved Institutional Animal Care And Use Committee (NCI IACUC) protocol (LBMB-001-4) of the National Cancer Institute, which is an Association for Assessment and Accreditation of Laboratory Animal Care International (AAALAC)–accredited research facility.

### Assembly of interphase nuclei and mitotic chromosomes in *Xenopus* egg extracts

Metaphase-arrested *X. laevis* egg extract was prepared as previously described (*59*). To examine de novo kinetochore assembly, *X. laevis* sperm nuclei and CaCl_2_ were added to the egg extract to release it into interphase and incubated for 90 min at 21°C. Reactions were then driven into M phase by dilution with two volumes of CSF-arrested extract and incubated for another 40 to 45 min at 21°C. Interphase nuclei or mitotic chromosomes were collected at various time points as indicated and processed for immunofluorescence staining.

### Immunodepletion of *Xenopus* egg extracts

Immunodepletions were performed using antibodies bound to Protein A beads (Dynabeads; Invitrogen). For immunodepletion of CENP-C or HJURP, 100 μl of extract was incubated for one or two rounds with 30 μg of anti–CENP-C or anti-HJURP antibody (*29*, *60*) bound to 100 μl of beads on ice for 45 min. For immunodepletion of CENP-T, 100 μl of extract was incubated for two rounds with 15 μg of anti–CENP-T antibody (*60*) bound to 100 μl of beads on ice for 45 min. For mock depletions, 100 μl of extract was incubated for two rounds with 10 μg of immunoglobulin G (IgG) (Sigma-Aldrich, I5006).

### Expression of exogenous mRNA in egg extracts

3xMyc–CENP-A and HJURP mRNAs were transcribed in vitro by using the mMessage Machine SP6 kit (Thermo Fisher Scientific). To prevent the translation of endogenous RNAs, RNase A (Ambion) was added to the extract at a final concentration of 100 ng/ml for 15 min at 12°C. An RNase inhibitor (RNasin Plus RNase Inhibitor; Promega) was added at a 1:50 dilution for 5 min at 12°C. Yeast tRNAs (50 μg/ml) were added, and the extract was kept on ice.

### DNA replication assay in *Xenopus* egg extracts

To study DNA replication, dimethyl sulfoxide (DMSO) or aphidicolin (100 μg/ml) (Sigma-Aldrich, A0781) was added to extracts supplemented with 10 μM Cy3-dUTP (Cytiva, PA53022). Then, sperm nuclei and CaCl_2_ were added to the reactions and incubated at 21°C for 90 min and then processed for fluorescence imaging.

### CENP-A loading assay in *Xenopus* egg extracts

To monitor the assembly of new CENP-A molecules at centromeres, 500 ng of 3xMyc–CENP-A and 500 ng of HJURP mRNAs were added per 20 μl of CSF-arrested extract and incubated at 21°C for 30 min to translate the 3xMyc–CENP-A and HJURP, as described previously (*7*). Cycloheximide was then added to the reactions to block further protein translation, followed by the addition of sperm nuclei and CaCl_2_ to release into interphase. The reactions were then incubated for 90 min at 21°C. Reactions were then processed for immunofluorescence. For experiments to examine the effects of PLK1 inhibition on CENP-A loading, DMSO or 10 μM BI-2536 (Selleckchem, S1109) was added to the indicated reactions 30 min after the addition of CaCl_2_ to allow for exit from M phase (*61*).

### Immunofluorescence and wide-field microscopy

To immunostain kinetochores, *Xenopus* egg extract was fixed for 5 min by 20-fold dilution in BRB80, 20% glycerol, 0.5% Triton X-100, and 3.7% formaldehyde at room temperature. Fixed reactions were layered onto a cushion of BRB80 and 40% glycerol overlaying poly-l-lysine–coated coverslips (No. 1) placed in a 24-well plate. Nuclei were adhered onto coverslips on plate holders at 4000 rpm for 15 min at 18°C in a centrifuge (Eppendorf 5810R). Cushions were washed with BRB80. For immunostaining of CENP-A, CENP-C, and CENP-O, coverslips were postfixed in ice-cold methanol for 5 min, blocked with Abdil [tris-buffered saline, 0.1% Tween 20, 2% bovine serum albumin (BSA), and 0.1% sodium azide] overnight at 4°C then incubated in the primary antibody at room temperature for 1 hour followed by secondary antibody (Alexa Fluor 488) at room temperature for 1 hour unless otherwise noted. For any immunostaining involving CENP-T, no methanol fixation was used, and Abdil with 3% BSA was used. For coimmunostaining of CENP-T or CENP-C and Myc-tagged or LAP-tagged proteins, coverslips were blocked with Abdil with 3% BSA at room temperature for 1.5 hours, incubated with Myc primary antibody or GFP-Booster Atto 488 at 18°C overnight, and then incubated with secondary antibody for 1 hour at room temperature. Coverslips were then incubated successively with CENP-T or CENP-C primary and secondary antibodies for 1 hour at room temperature. All washes and antibody dilutions were done with AbDil buffer. Nuclei were stained with Hoechst 33258 unless otherwise noted. Coverslips were mounted on a glass slide in 80% glycerol in a phosphate-buffered saline (PBS) medium. The following antibodies were used at the indicated dilutions: CENP-C (*60*), 1:150; CENP-T (*60*), 1:100; CENP-A (*62*) (a gift from A. Straight, Stanford University), 1:500; CENP-O (a gift from A. Arnoutov and M. Dasso, NICHD, NIH), 1:200; Histone H3 (Thermo Fisher Scientific, 865R2) 1:250; anti-Myc (Santa Cruz, sc-40), 1:100; GFP-Booster Atto 488 (ChromoTek, gba488) 1:400; Alexa Fluor 488 (Jackson ImmunoResearch, 711-545-152), 1:400; and Alexa Fluor 647 (Jackson ImmunoResearch, 711-605-152), 1:400. For quantification of immunofluorescence, samples were imaged with a 0.2- or 0.4-μm step size using an Eclipse Ti (Nikon) composed of a Nikon Plan Apo 100×/1.45, oil immersion objective, a Plan Apo 40×/0.95 objective, and a Hamamatsu Orca-Flash 4.0 camera. Images were captured and processed using Nikon’s NIS Elements AR 4.20.02 software and analyzed in ImageJ/Fiji. The acquired *Z*-sections were converted into a maximum intensity projection using NIS Elements and Fiji. Kinetochore intensity was measured using Fiji centering regions of 9 × 9 and 13 × 13 pixels over individual kinetochores. Total fluorescence intensity was recorded from each region. To correct for background fluorescence, the intensity difference between the two regions was determined and then scaled to the smaller region. This background value was then subtracted from the smaller region to determine kinetochore intensity with background correction, as previously reported (*63*).

### Interphase progression assay in *Xenopus* egg extracts

To study the kinetics of interphase progression, sperm nuclei and CaCl_2_ were added to CSF-arrested extract and incubated at 21°C for 90 min. To examine the effects of PLK1 inhibition on cell progression, DMSO or 10 μM BI-2536 was added to the indicated reactions 30 min after the addition of CaCl_2_. The samples were collected at the indicated time points. Reactions were then processed for immunofluorescence. The nuclear cross-sectional area was measured in ImageJ/Fiji by thresholding in the DNA channel. Time points between *t* = 30′ and *t* = 90′ were fit with an exponential growth model in GraphPad Prism.

### Mouse strains

CF1 mice (Non-Swiss Albino, stock #033) were purchased from Envigo. Mouse experiments were approved by the Animal Care and Use Committee (National Institutes of Health Animal Study Proposal #H-0327) and were consistent with the National Institutes of Health guidelines.

### Granulosa cell culture and immunostaining

The procedure for isolating and culturing ovarian granulosa cells has been described previously (*64*). Briefly, after euthanizing the mice, their ovaries were collected and rinsed three times with M2 media (Sigma-Aldrich, catalog no. M7167) to remove any adherent fat tissue. The ovaries were then mechanically disrupted to release oocytes and granulosa cells. Following oocyte collection, the remaining granulosa cells were collected into a 15-ml tube and allowed to settle for 5 to 10 min. The supernatant was discarded to remove blood cells, and the granulosa cells were then centrifuged at 500*g* for 5 min. The cells were washed extensively with Dulbecco’s modified Eagle’s medium (DMEM) high-glucose GlutaMAX media (Gibco, catalog no. 10566-016) supplemented with 1× antibiotic-antimycotic (Gibco, catalog no. 15240062). Cells were dispersed by pipetting, washed two additional times, seeded on a 6-well tissue culture plate (Falcon, catalog no. 353046) with DMEM supplemented with 10% fetal bovine serum (Gibco, catalog no. A3160501) and 1× antibiotic-antimycotic, and cultured in a humidified atmosphere containing 5% CO_2_ at 37°C. After 24 hours, the medium was replaced with fresh media of the same type to continue the primary culture for 4 days. To suspend the cells, the culture was treated with 0.25% trypsin-EDTA (Gibco, catalog no. 25200-056) for 5 min at room temperature and collected in a 15-ml tube and centrifuged at 500*g* for 5 min. The cells were washed three times with 1x PBS. The cell nuclei were then spread on poly-l-lysine–coated coverslips (No. 1) with 1% paraformaldehyde (Electron Microscopy Sciences, catalog no. 15710), 0.15% Triton X-100 (Millipore, catalog no. TX1568-1), and 3 mM dithiothreitol (Sigma-Aldrich, catalog no. 43815), overnight in a humid chamber to ensure that the cell nuclei attach securely to the coverslip. After air drying, the cells were washed with 1× PBS to remove the fixative solution, treated with λ-phosphatase (NEB, catalog no. P0753L) for 1 hour at 30°C, placed in the blocking solution [0.3% BSA (Fisher Bioreagents, catalog no. BP1600-100) and 0.01% Tween 20 (Thermo Fisher Scientific, catalog no. J20605-AP) in 1× PBS] 10 min at room temperature, incubated for 2 hours with the CENP-A (1:100, Cell Signaling, catalog no. 2048) primary antibody at room temperature, washed three times for 10 min with the blocking solution, incubated for 1 hour with Alexa Fluor 488–conjugated donkey anti-rabbit secondary antibody (1:500; Invitrogen, catalog no. A21206) at room temperature, washed three times for 10 min in the blocking solution, and mounted on microscope slides with the Antifade Mounting Medium with 4′,6-diamidino-2-phenylindole (DAPI; Vector Laboratories, catalog no. H-1200).

### Super-resolution microscopy data acquisition and reconstruction

Unless otherwise noted, 3D-SIM imaging was performed on a GE DeltaVision OMX SR microscope (GE Healthcare) equipped with a 60x 1.42–numerical aperture (NA) oil objective (Olympus) using DeltaVision immersion oil with a refractive index of 1.514. *Z*-stacks were acquired for a single channel at 0.125-μm increments. Raw data were reconstructed using the SoftWorx software (GE Healthcare) with a Wiener filter constant set to 0.003. Lasers with wavelengths of 405, 488, and 640 nm were used to image DAPI, Alexa Fluor 488, or GFP-Booster Atto 488 and Alexa Fluor 647.

SIM super-resolution images (fig. S1G, left) were acquired using a Zeiss LSM780 Elyra SIM microscope equipped with a 63x plan-apochromat (NA 1.4) oil immersion objective lens. Images were collected with a 0.032-μm *x*-*y* pixel size, a *z*-step size of 0.090 μm, five phases, and three rotations of the SIM grating. Images were processed using the SIM image processing algorithm in the Zeiss Zen software (version 13.0). A 488-nm laser was used to image Alexa Fluor 488.

Airyscan super-resolution images (fig. S1G, right) were acquired using a Zeiss LSM880 microscope equipped with a 63x plan-apochromat (NA 1.4) oil immersion objective lens. Images were collected with a 0.044-μm *x*-*y* pixel size and a 0.160-μm *z*-step size. The images were processed using the Airyscan processing algorithm in the Zeiss Zen software (version 14.0.18). A 488-nm laser was used to image Alexa Fluor 488.

Nikon SoRa super-resolution images (fig. S1G, middle) were acquired using a Nikon SoRa spinning disk microscope equipped with a 60x Apo TIRF (NA 1.49) oil immersion objective lens and Photometrics BSI sCMOS camera. Images were collected with a 0.03-μm *x*-*y* pixel size and a 0.15-μm *z*-step size and deconvolved using a modified Lucy-Richardson constrained iterative deconvolution algorithm in the Nikon Elements image analysis software (version 5.4.1). A 488-nm laser was used to image Alexa Fluor 488.

Hessian 3D-SIM super-resolution (*65*) images (Fig. 1E) were acquired using a Cell Xtreme microscope from CSR Biotech, equipped with a 100x (NA 1.5) oil immersion Nikon objective. *Z*-stacks were acquired for a single channel at 0.2-μm increments. Raw data were processed using a sparse deconvolution algorithm (*65*). Lasers with 405- and 488-nm wavelengths were used to image DAPI and Alexa Fluor 488.

### 3D-SIM image analysis

ImageJ/Fiji was used for the 3D-SIM image analysis. A single plane from a *z*-stack of a single-channel or double-channel image stack was used for image analysis and visualization. Unless otherwise noted, a representative image of a single kinetochore was cropped and shown in the figures. Wide-field-equivalent images [Fig. 1D (top) and fig. S1A] were generated from the raw SIM data by Fourier filtering in Fiji to suppress SIM/moiré components. These SIM-derived pseudo-wide-field images are shown at the same magnification and field of view as the corresponding reconstructed SIM images. An orthogonal view (*XZ* or *YZ*) of an *XY* plane was created using Fiji’s orthogonal views tool. The line intensity profile across the kinetochore structure was measured using Fiji’s plot profile tool. The mean background intensity for centromeres and kinetochores was measured from the region within the nucleus that lacked a centromere or kinetochore, whereas for DAPI, it was estimated from the exterior of the nucleus. The intensity profile of a single channel was generated by subtracting the mean background intensity per pixel from the total intensity per pixel along a line intensity profile. The background-corrected intensity profile was then normalized to the maximum intensity, and the minimum or maximum peak of the intensity profile was recorded as a center of the curve and plotted against the distance from the center. The maximum width of a kinetochore ring was defined by measuring the distance between the two highest peaks in the intensity profile generated from a line scan across the longest axis of the ring structure.

### Deep learning analysis of 3D-SIM data

Using neural networks to improve axial resolution was described previously (*26*). Model training and model application were performed on an NVIDIA RTX A6000 GPU (with 48-GB memory) installed on a local workstation. We used Python version 3.9.18 for all neural networks. For training, we first interpolated the 3D-SIM volumes to 40-nm pixel size in all three dimensions. The interpolated volumes were blurred with a 1D Gaussian function, sigma = 2.4 pixels along the *x* dimension. Next, the interpolated volumes were blurred with a 1D Gaussian function (sigma = 1.0 pixel) along the *z* dimension to remove spurious sidelobes in the Fourier domain. The purpose of these blurring operations is to degrade lateral resolution so it resembles the native axial resolution of the 3D-SIM system. Stacks were then downsampled along the *x* dimension to mimic the coarse axial sampling of the experiment and upsampled to recover an isotropic pixel size, serving as the “input” for training for the CARE network (*66*). The goal of the network is to reverse this image degradation, thereby improving resolution along the degraded *x* direction. Patches of size 64 × 64 × 64 were randomly cropped from the data, and 15% of the patches were set aside for validation. The training learning rate was 2 × 10^4^; the number of epochs was 100; the number of steps per epoch was 200; and the mean absolute error (MAE) was used as loss function. To apply the trained model, we (i) interpolated, blurred, downsampled, and upsampled 3D-SIM volumes as described above; (ii) rotated the resulting volumes at six distinct angles about the “*y*” axis; (iii) applied the network to each rotated volume to increase resolution in 1D; (iv) rotated the network predictions back to their original orientation; (v) cropped them to their original size; (vi) Fourier transformed each volume; (vii) computed the maximum value at each spatial frequency across all rotations; and (viii) obtained the final result by applying inverse Fourier transform and taking the absolute value.

### In vitro transcription and translation

The SP6-coupled in vitro transcription and translation (TNT) system (Promega) was used to express CENP-T–LAP, CENP-C–LAP, HJURP-LAP, Mis18ɑ-LAP, Mis18β-LAP, and M18BP1-LAP from 2 μg of plasmid DNA. To assess the localization of LAP-tagged protein, 2 μl of the TNT reaction was added to 20 μl of the indicated extract condition at the beginning of interphase.

### Purification of GST-HJURP^1-100^ and GST

GST-HJURP^1-100^ or GST was expressed in BL21 (DE3) Rosetta-2 cells. Expression was induced with 0.5 mM isopropyl-β-d-thiogalactopyranoside for 2.5 hours at 37°C. Lysates containing GST-HJURP^1-100^ or GST were bound to Glutathione Sepharose 4B resin and eluted with 20 mM Hepes, 20 mM reduced glutathione (pH 7.9), 500 mM NaCl, and 1 mM TCEP and exchanged into XB buffer [0.1 M KCl, 0.1 mM MgCl_2_, 50 mM sucrose, 10 mM Hepes (pH 7.7), and 1 mM TCEP]. GST-HJURP^1-100^ or GST was added to the extract at a final concentration of 5 μM.

### Coimmunoprecipitations

Immunoprecipitations were performed to determine the binding of GST-HJURP^1-100^ to soluble CENP-A in high-speed supernatant (HSS) *Xenopus* egg extracts, which were prepared as described previously (*67*). Purified GST-HJURP^1-100^ or GST was added to 50 μl of HSS extracts without chromatin to a final concentration of 5 μM and incubated at 21°C for 30 min. Fifty microliters of Glutathione Sepharose 4B beads was washed four times in binding buffer [20 mM Hepes (pH 7.7), 150 mM NaCl, and 1 mM TCEP] and added to the extracts and then incubated with end-over-end rotation for 1 hour at 4°C. Beads were briefly spun and washed four times with wash buffer [20 mM Hepes (pH 7.7), 150 mM NaCl, 1 mM TCEP, and 0.01% NP-40] before eluting with 2X sample buffer.

### Western blots

Primary antibodies were diluted in LI-COR blocking solution with a final Tween 20 concentration of 0.1%. The following antibodies and antibody dilutions were used: anti–CENP-T, 1:250; anti–CENP-C 1:250; anti-HJURP 1:200; GST (Pierce, 700775) 1:1000; anti–CENP-A serum (this paper) 1:100; anti-histone H3T3ph (Epitomics, 2162-1) 1:10,000; anti-Myc (Santa Cruz, sc-40), 1:00; and anti-α-tubulin (DM1, 1:20,000; Sigma-Aldrich). Secondary antibodies from LI-COR were used (goat anti-rabbit, 800 nm; goat anti-mouse, 680 nm), as was the LI-COR imaging system to scan membranes.

### Quantification and statistical calculation

All image analysis of centromeres and kinetochores was performed using a minimum of 50 structures per assay. Sample size was chosen to ensure a high (>90%) theoretical statistical power to generate reliable *P* values. A *P* value of <0.05 was considered significant. Statistical details of the experiments can be found in the figures, figure legends, and results sections. All graphs and statistical analyses were prepared using GraphPad Prism, in which each dataset was visualized as SuperPlots (*68*). Specifically, each biological replicate is color coded, with individual measurements and the replicate mean shown. Combined datasets from replicated experimental conditions were compared to control conditions using an ordinary one-way analysis of variance (ANOVA) with Tukey’s multiple comparison tests to determine significance. The biological replicates in the quantifications in Figs. 2B and 3G and figs. S5H and S7H were acquired using two exposure-time settings; all intensity measurements were background subtracted and normalized to the matched control within each exposure-matched replicate before pooling. All graphs show the mean with error bars representing the SD of the aggregate of individual measurements unless otherwise indicated. At least two biological replicates (using different preparations of egg extracts or cells) were performed for each experiment, and each confirmed similar behavior among the replicates.
