# Supplementary material for: The CENP-A chaperone complex spatially organizes centromeres
Source: Sci Adv. 2026 Sep 9;12(37):eaef1579. doi: 10.1126/sciadv.aef1579 (PMC13557081; doi:10.1126/sciadv.aef1579)
Supplement: Supplementary file 1 [file sciadv.aef1579_sm.pdf]

Science Advances
AAAS

# Supplementary Materials for

## The CENP-A chaperone complex spatially organizes centromeres

Hindol Gupta *et al.*

Corresponding author: Alexander E. Kelly, alexander.kelly@nih.gov

*Sci. Adv.* **12**, eaef1579 (2026)
DOI: 10.1126/sciadv.aef1579

**This PDF file includes:**

Figs. S1 to S7

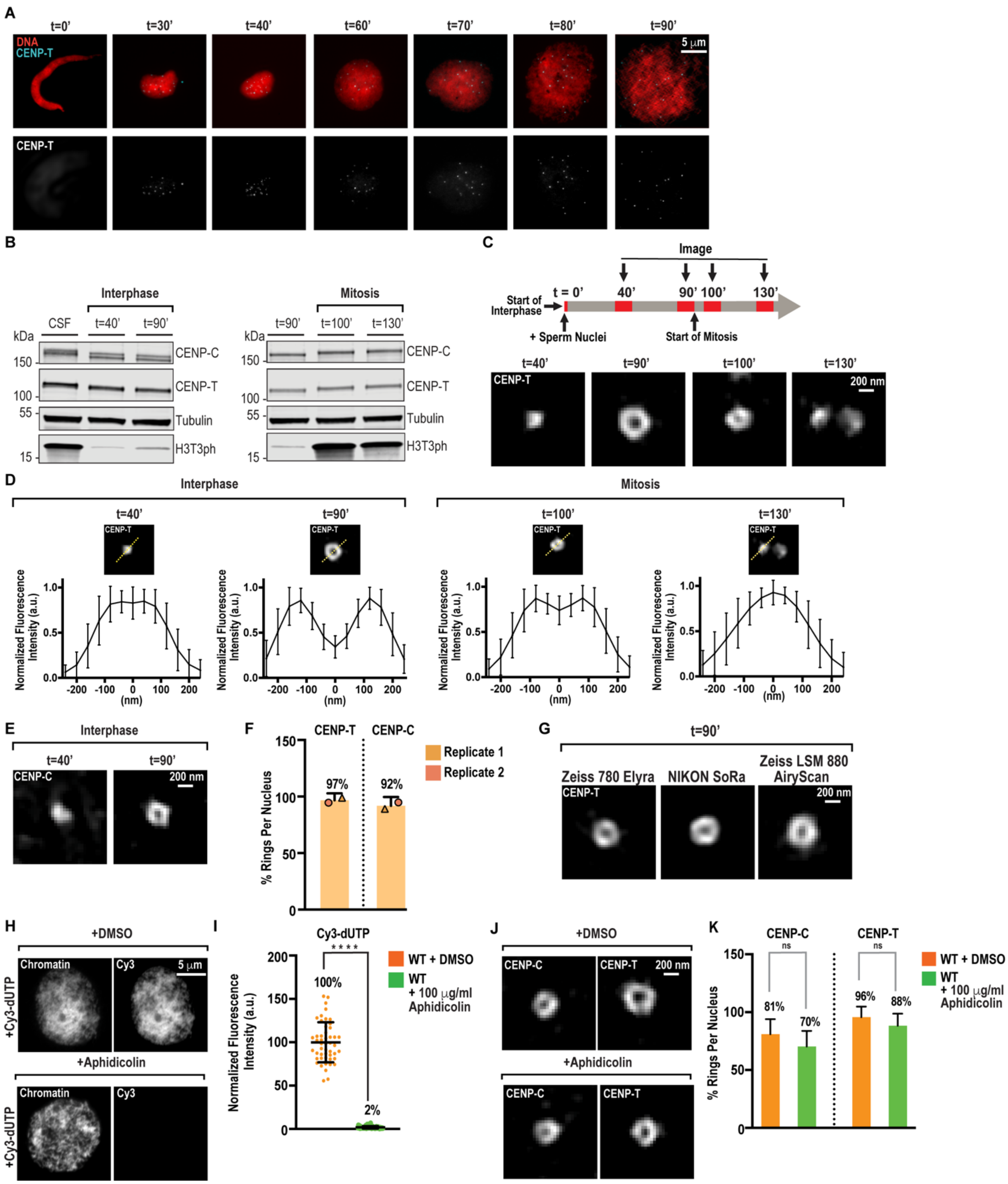

**Fig. S1. Centromeric chromatin architecture changes during the cell cycle but is independent of DNA replication.** (**A**) Top, representative SIM-derived widefield-equivalent merged images of interphase nuclei stained for CENP-T (cyan) and DNA (red) at the indicated times, generated from the raw SIM data by Fourier filtering; bottom, CENP-T alone. Scale bar, 5 µm. (**B**) Western blot for CENP-C, CENP-T, tubulin, and histone H3 phosphorylation (H3T3ph)

for the samples in (C). CSF (cytostatic factor) *Xenopus* egg extracts are arrested in M phase without prior cycling through interphase. (**C**) Top, timeline for imaging kinetochores on interphase nuclei and mitotic chromosomes in *Xenopus* egg extract; samples were collected at the indicated times (in minutes) after adding sperm and $Ca^{2+}$ and processed for immunofluorescence. Bottom, representative 3D-SIM images of CENP-T at the indicated timepoints. (**D**) Averaged and aligned linescans of CENP-T at kinetochores from (C). Each individual trace (not shown) was aligned to its central minimum or maximum and normalized to its maximum, and the average trace plotted on this scale. Insets illustrate the linescan method. n = 75 kinetochores per condition (≥ 6 nuclei). (**E**) Representative 3D-SIM images of CENP-C in interphase nuclei ($t$ = 40′ and 90′) from WT extract. Scale bar, 200 nm. (**F**) Percentage of observable CENP-C and CENP-T ring-like structures per nucleus. SuperPlot of two biological replicates, replicate means color-coded. n = 10 nuclei per condition (≥ 145 kinetochores). Error bars, SD. (**G**) Representative super-resolution images of CENP-T in interphase nuclei ($t$ = 90′) from WT extract, acquired on the indicated microscope platforms. Scale bar, 200 nm. (**H**) Representative immunofluorescence of chromatin (DNA, Hoechst) and Cy3-dUTP in interphase nuclei ($t$ = 90′), assembled from extract treated with DMSO or aphidicolin. Scale bar, 5 µm. (**I**) Cy3-dUTP fluorescence intensity from (H), normalized to DMSO. n = 50 nuclei per condition. A.U., arbitrary units. Error bars, SD; ****, $P < 0.0001$. (**J**) Representative 3D-SIM images of CENP-C and CENP-T in interphase nuclei ($t$ = 90′), assembled from extract treated with DMSO or aphidicolin. Scale bar, 200 nm. (**K**) Percentage of CENP-C and CENP-T rings per nucleus after DMSO or aphidicolin treatment, for (J). n = 10 nuclei per condition (≥ 145 centromeres). Error bars, SD; ns, not significant.

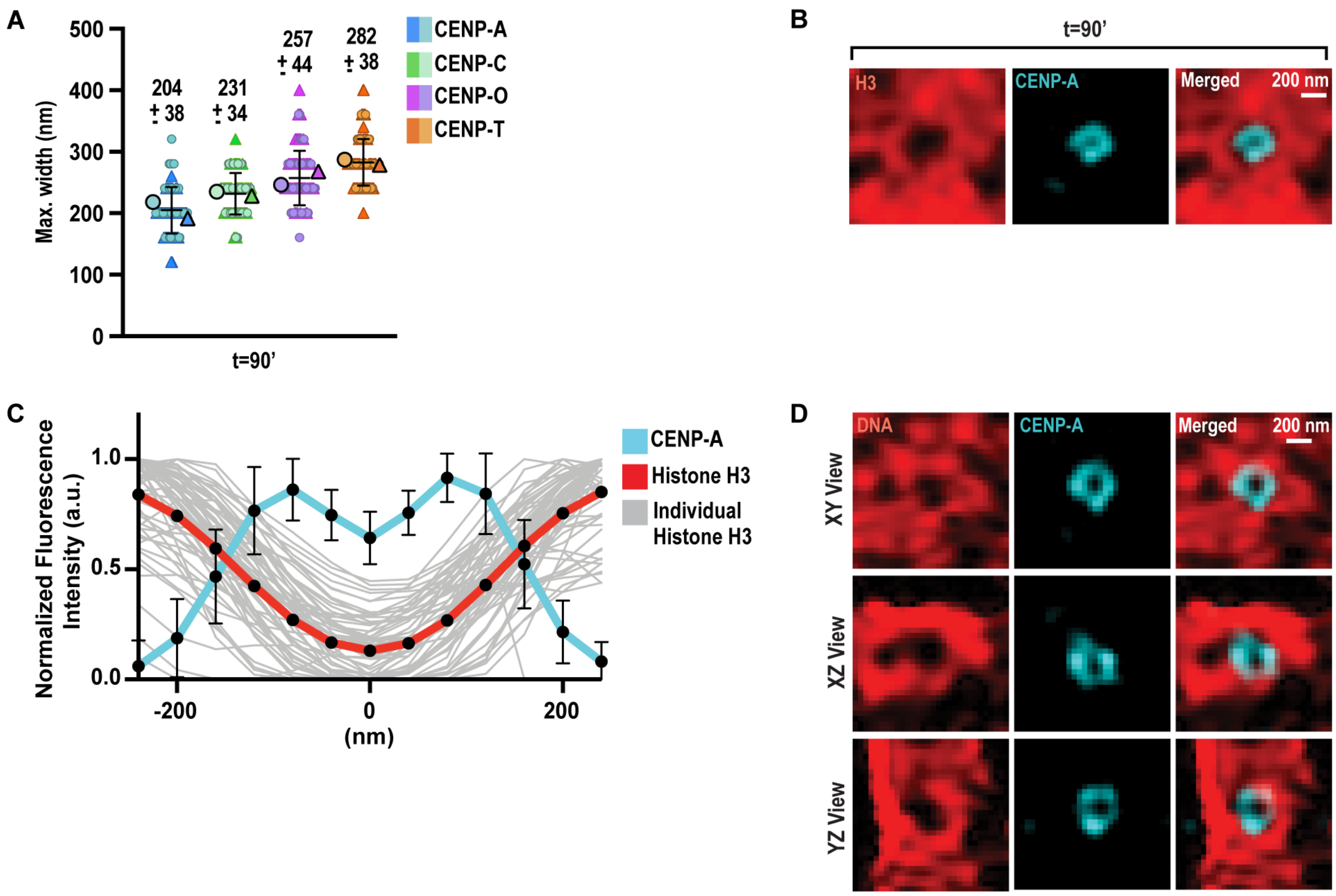


**Fig. S2. 3D-SIM and deep learning analysis of CCAN and CENP-A structures.** (**A**) Average peak-to-peak distances of CENP-A, CENP-C, CENP-O, and CENP-T signals from linescans of 3D-SIM images of interphase nuclei ($t$ = 90′); representative images are in Fig. 1D. SuperPlot of two biological replicates, replicate means color-coded. n = 75 centromeres or kinetochores per condition (≥ 5 nuclei). Error bars, SD. (**B**) Representative 3D-SIM image of histone H3 (red) and CENP-A (cyan) in interphase nuclei ($t$ = 90′), showing the individual and merged channels. Scale bar, 200 nm. (**C**) Linescans of histone H3 and CENP-A at centromeres from (B). Bold lines, mean intensity; thin gray lines, individual histone H3 traces, each aligned to the central minimum and normalized to its maximum. n = 60 centromeres, 5 nuclei. Error bars, SD. (**D**) Axial resolution enhancement of 3D-SIM images of CENP-A (cyan) and DNA (DAPI, red) by deep-learning prediction. Representative XY, XZ, and YZ views are shown. Scale bar, 200 nm.

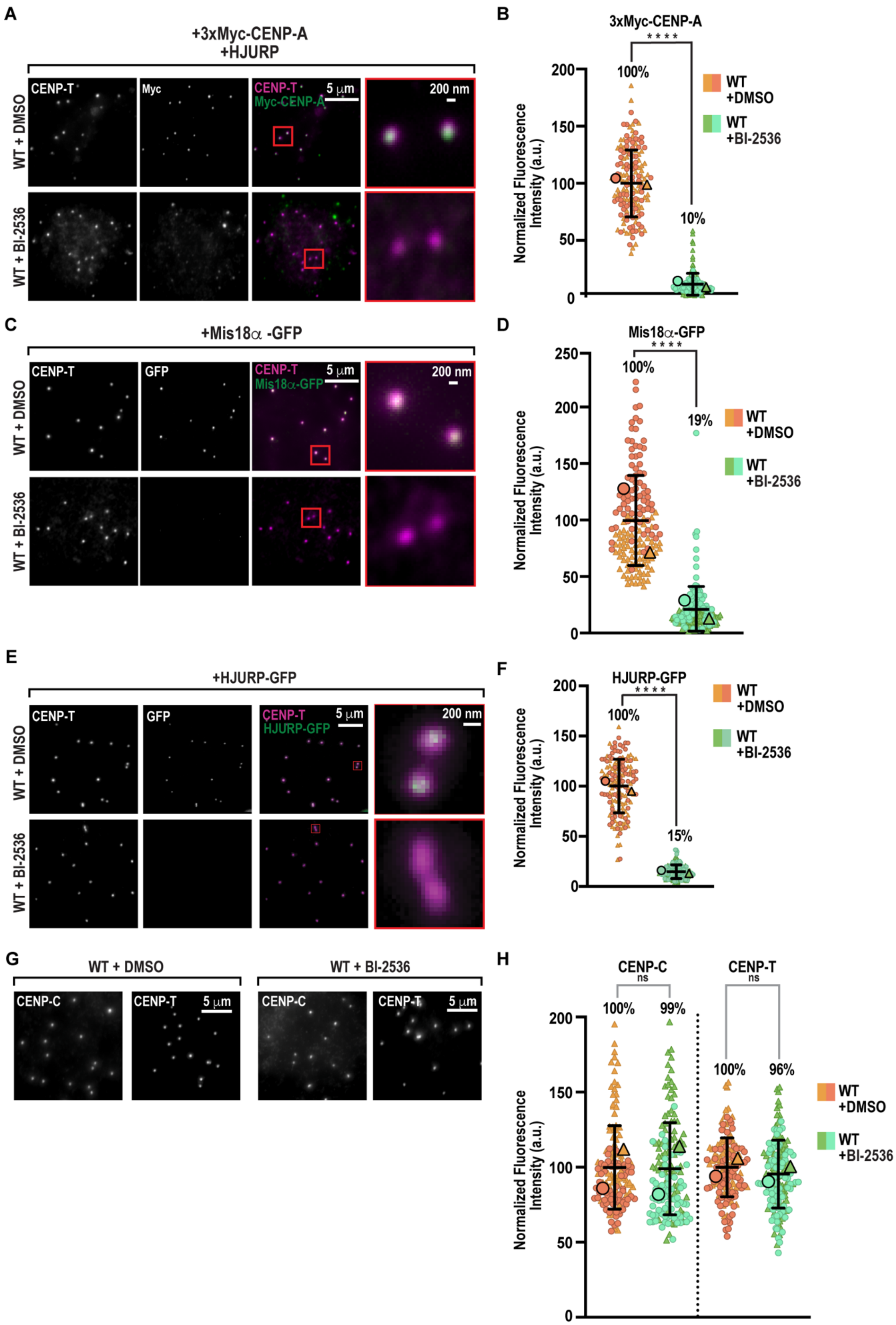
A
+3xMyc-CENP-A
+HJURP
CENP-T
Myc
CENP-T
Myc-CENP-A
5 μm
200 nm
WT + DMSO
WT + BI-2536
B
3xMyc-CENP-A
****
100%
10%
Normalized Fluorescence Intensity (a.u.)
WT +DMSO
WT +BI-2536
C
+Mis18α -GFP
CENP-T
GFP
CENP-T
Mis18α-GFP
5 μm
200 nm
WT + DMSO
WT + BI-2536
D
Mis18α-GFP
****
100%
19%
Normalized Fluorescence Intensity (a.u.)
WT +DMSO
WT +BI-2536
E
+HJURP-GFP
CENP-T
GFP
CENP-T
HJURP-GFP
5 μm
200 nm
WT + DMSO
WT + BI-2536
F
HJURP-GFP
****
100%
15%
Normalized Fluorescence Intensity (a.u.)
WT +DMSO
WT +BI-2536
G
WT + DMSO
WT + BI-2536
CENP-C
CENP-T
5 μm
H
CENP-C
ns
CENP-T
ns
100%
99%
100%
96%
Normalized Fluorescence Intensity (a.u.)
WT +DMSO
WT +BI-2536

**Fig. S3. Plk1 inhibition blocks new CENP-A assembly and chaperone localization in *Xenopus* egg extracts.** (**A**) Representative immunofluorescence of CENP-T (magenta) and 3XMyc-CENP-A (green) in interphase nuclei ($t$ = 90′), assembled from extract containing 3XMyc-CENP-A and HJURP mRNA and treated with DMSO or BI-2536. Red boxed regions are magnified in the last column. Scale bars, 5 µm (main) and 200 nm (magnified). (**B**) 3XMyc-CENP-A fluorescence intensity from (A), normalized to DMSO. SuperPlot of two biological replicates, replicate means color-coded. n = 75 kinetochores per condition (≥ 6 nuclei). A.U., arbitrary units. Error bars, SD; ****, $P < 0.0001$. (**C**) Representative immunofluorescence of CENP-T (magenta) and Mis18α-GFP (green) in interphase nuclei ($t$ = 90′), assembled from extract containing *in vitro*-translated Mis18α-GFP and treated with DMSO or BI-2536. Red boxed regions are magnified in the last column. Scale bars, 5 µm (main) and 200 nm (magnified). (**D**) Mis18α-GFP fluorescence intensity from (C), normalized to DMSO. SuperPlot of two biological replicates, replicate means color-coded. n = 75 kinetochores per condition (≥ 6 nuclei). A.U., arbitrary units. Error bars, SD; ****, $P < 0.0001$. (**E**) Representative immunofluorescence of CENP-T (magenta) and HJURP-GFP (green) in interphase nuclei ($t$ = 90′), assembled from extract containing *in vitro*-translated HJURP-GFP and treated with DMSO or BI-2536. Red boxed regions are magnified in the last column. Scale bars, 5 µm (main) and 200 nm (magnified). (**F**) HJURP-GFP fluorescence intensity from (E), normalized to DMSO. SuperPlot of two biological replicates, replicate means color-coded. n = 75 kinetochores per condition (≥ 6 nuclei). A.U., arbitrary units. Error bars, SD; ****, $P < 0.0001$. (**G**) Representative immunofluorescence of CENP-C and CENP-T in interphase nuclei ($t$ = 90′), assembled from extract treated with DMSO or BI-2536. Scale bar, 5 µm. (**H**) CENP-C and CENP-T fluorescence intensities from (G), normalized to DMSO. SuperPlot of two biological replicates, replicate means color-coded. n = 75 kinetochores per condition (≥ 6 nuclei). A.U., arbitrary units. Error bars, SD; ns, not significant.

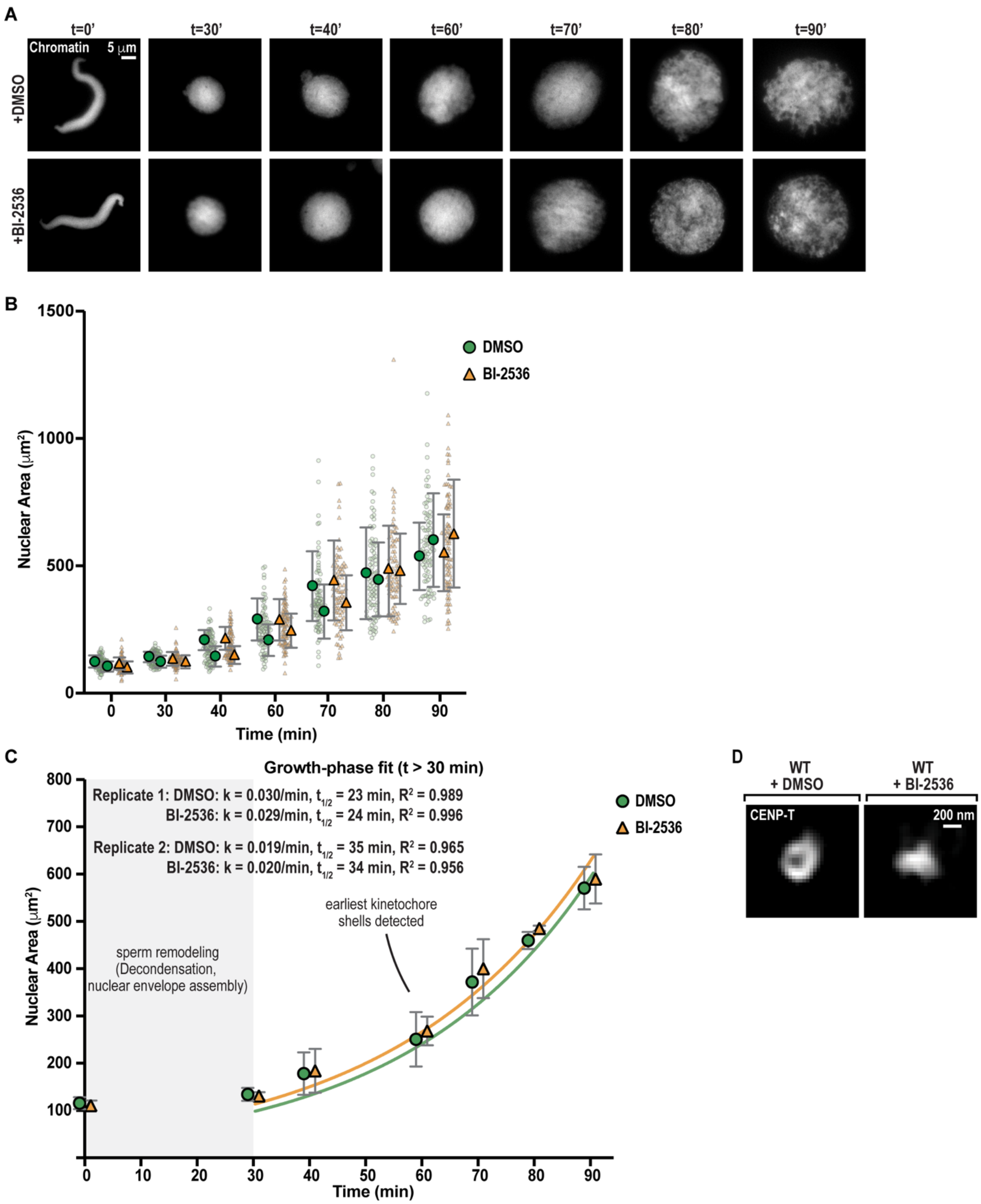


**Fig. S4. Plk1 inhibition does not impede or delay interphase progression.**
(**A**) Representative immunofluorescence of DNA (Hoechst) at the indicated times in interphase nuclei assembled from extract treated with DMSO or BI-2536 at *t* = 30′. Scale bar, 5 µm. (**B**) Cross-sectional nuclear area (µm$^2$) from (A). SuperPlot of two biological replicates, replicate

means color-coded. n = 45 nuclei per condition. Error bars, SD. (**C**) Single-exponential growth fits ($Y = Y_0e^{kt}$) of the data in (B), shown as mean ± SD across both replicates. The shaded interval ($t$ = 0 to 30′) marks the remodeling/nuclear-envelope-assembly phase, when elongated sperm chromatin is converted into rounded interphase nuclei; $t$ = 0 was excluded from fitting because elongated sperm nuclei are morphologically distinct and not comparable in area. Fits used the growth phase ($t \geq 30′$) of one replicate; fitted rate constants and doubling times for each replicate and condition are inset, and fits are comparable between conditions. (**D**) Representative 3D-SIM images of CENP-T in interphase nuclei ($t$ = 90′) from the samples in (A)

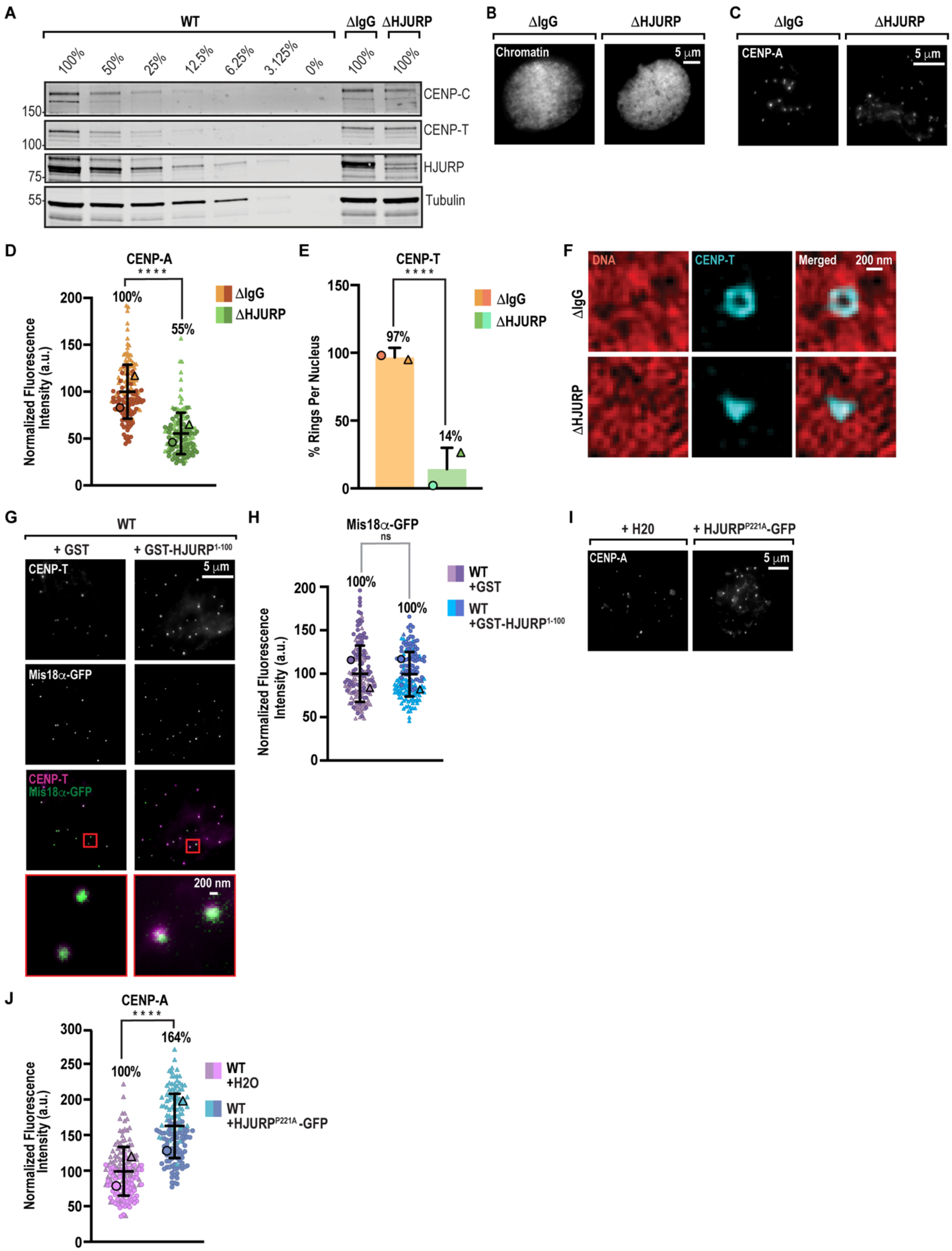

A
WT
ΔIgG
ΔHJURP
100%
50%
25%
12.5%
6.25%
3.125%
0%
100%
100%
CENP-C
CENP-T
HJURP
Tubulin
150
100
75
55
B
ΔIgG
ΔHJURP
Chromatin
5 μm
C
ΔIgG
ΔHJURP
CENP-A
5 μm
D
CENP-A
****
100%
55%
ΔIgG
ΔHJURP
Normalized Fluorescence Intensity (a.u.)
200
150
100
50
0
E
CENP-T
****
97%
14%
ΔIgG
ΔHJURP
% Rings Per Nucleus
150
100
50
0
F
DNA
CENP-T
Merged
200 nm
ΔIgG
ΔHJURP
G
WT
+ GST
+ GST-HJURP1-100
CENP-T
5 μm
Mis18α-GFP
CENP-T
Mis18α-GFP
200 nm
H
Mis18α-GFP
ns
100%
100%
WT +GST
WT +GST-HJURP1-100
Normalized Fluorescence Intensity (a.u.)
200
150
100
50
0
I
+ H20
+ HJURPP221A-GFP
CENP-A
5 μm
J
CENP-A
****
100%
164%
WT +H2O
WT +HJURPP221A-GFP
Normalized Fluorescence Intensity (a.u.)
300
250
200
150
100
50
0

**Fig. S5. HJURP, but not its role in CENP-A assembly, is required for centromere organization in interphase.** (**A**) Western blot for CENP-C, CENP-T, HJURP, and tubulin in WT, ΔIgG, and ΔHJURP extracts from the samples in Fig. 3A. (**B**) Representative immunofluorescence of DNA (Hoechst) in interphase nuclei (*t* = 90′) from ΔIgG and ΔHJURP extracts. Average cross-sectional nuclear area, ΔIgG: 386 +/- 91 $\mu m^2$, ΔHJURP: 410 +/- 96 $\mu m^2$. n = 40 nuclei per condition. Scale bar, 5 µm. (**C**) Representative immunofluorescence of CENP-A in interphase nuclei (*t* = 90′) from ΔIgG and ΔHJURP extracts. Scale bar, 5 µm. (**D**) CENP-A fluorescence intensity from (C), normalized to ΔIgG. SuperPlot of two biological replicates, replicate means color-coded. n = 75 centromeres per condition (≥ 6 nuclei). A.U., arbitrary units. Error bars, SD; ****, $P < 0.0001$. (**E**) Percentage of observable CENP-T ring-like structures per nucleus for the conditions in Fig. 3A. SuperPlot of two biological replicates, replicate means color-coded. n = 10 nuclei per condition (≥ 145 kinetochores). Error bars, SD; ****, $P < 0.0001$. (**F**) Representative 3D-SIM images of CENP-T (cyan) and DNA (DAPI, red) in interphase nuclei (*t* = 90′) from ΔIgG or ΔHJURP extract, for Fig. 3 (B and C). Scale bar, 200 nm. (**G**) Representative immunofluorescence of CENP-T (magenta) and Mis18α-GFP (green) in interphase nuclei (*t* = 90′), assembled from extract containing *in vitro*-translated Mis18α-GFP and treated with GST or GST-$HJURP^{1\text{-}100}$. Red boxed regions are magnified in the last row. Scale bars, 5 µm (main) and 200 nm (magnified). (**H**) Mis18α-GFP fluorescence intensity from (G), normalized to GST. SuperPlot of two biological replicates, replicate means color-coded. n = 75 kinetochores per condition (≥ 6 nuclei). A.U., arbitrary units. Error bars, SD; ns, not significant. (**I**) Representative immunofluorescence of CENP-A in interphase nuclei (*t* = 90′) from extract with water ($H_2O$) or $HJURP^{P221A}$-GFP mRNA. Scale bar, 5 µm. (**J**) CENP-A fluorescence intensity from (I), normalized to $H_2O$. SuperPlot of two biological replicates, replicate means color-coded. n = 75 centromeres per condition (≥ 6 nuclei). A.U., arbitrary units. Error bars, SD; ****, $P < 0.0001$.

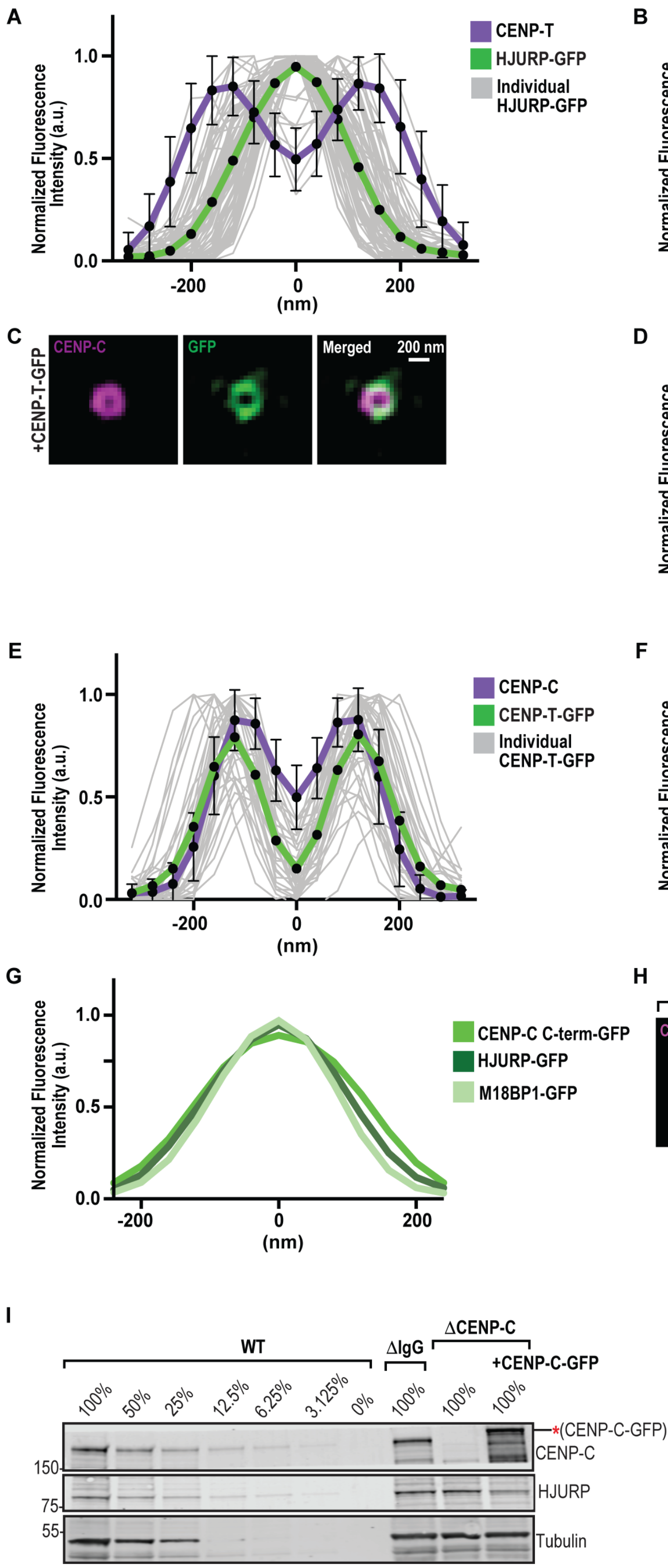


**Fig. S6. The CENP-A chaperone complex and the CENP-C C-terminus occupy the chromatin clearing.** (**A**) Linescans of CENP-T and HJURP-GFP at centromeres from Fig. 4A.

Bold lines, mean intensity; thin gray lines, individual HJURP-GFP traces, each aligned to the central minimum and normalized to its maximum. n = 75 kinetochores (≥ 6 nuclei). Error bars, SD. (**B**) Linescans of CENP-T and M18BP1-1-GFP at centromeres from Fig. 4A, analyzed as in (A). n = 75 kinetochores (≥ 6 nuclei). Error bars, SD. (**C**) Representative 3D-SIM images of CENP-C (magenta) and GFP (green) in interphase nuclei (*t* = 90′), assembled from extract containing *in vitro*-translated CENP-T-GFP. Scale bar, 200 nm. (**D**) Linescans of CENP-C and CENP-T-GFP at kinetochores from (C). Bold lines, mean intensity; thin gray lines, individual CENP-C traces, each aligned to the central minimum and normalized to its maximum. n = 35 kinetochores, 4 nuclei. Error bars, SD. (**E**) Linescans of CENP-C and CENP-T-GFP at centromeres from (C). Bold lines, mean intensity; thin gray lines, individual CENP-T-GFP traces, each aligned to the central minimum and normalized to its maximum. n = 35 kinetochores, 4 nuclei. Error bars, SD. (**F**) Linescans of CENP-T and CENP-C-GFP at centromeres from Fig. 4D. Bold lines, mean intensity; thin gray lines, individual CENP-C-GFP traces, each aligned to the central minimum and normalized to its maximum. n = 75 kinetochores, 6 nuclei. Error bars, SD. (**G**) Averaged, aligned linescans of CENP-C-GFP, HJURP-GFP, and M18BP1-GFP. (**H**) Representative 3D-SIM images of CENP-C (magenta) and GFP (green) in interphase nuclei (*t* = 90′), assembled from ΔCENP-C extract containing *in vitro*-translated CENP-C-GFP. Scale bar, 200 nm. (**I**) Western blot for CENP-C-GFP, CENP-C, HJURP, and tubulin in WT, ΔIgG, ΔCENP-C, and ΔCENP-C + CENP-C-GFP extract from the samples in (H). The asterisk indicates the CENP-C-GFP band.

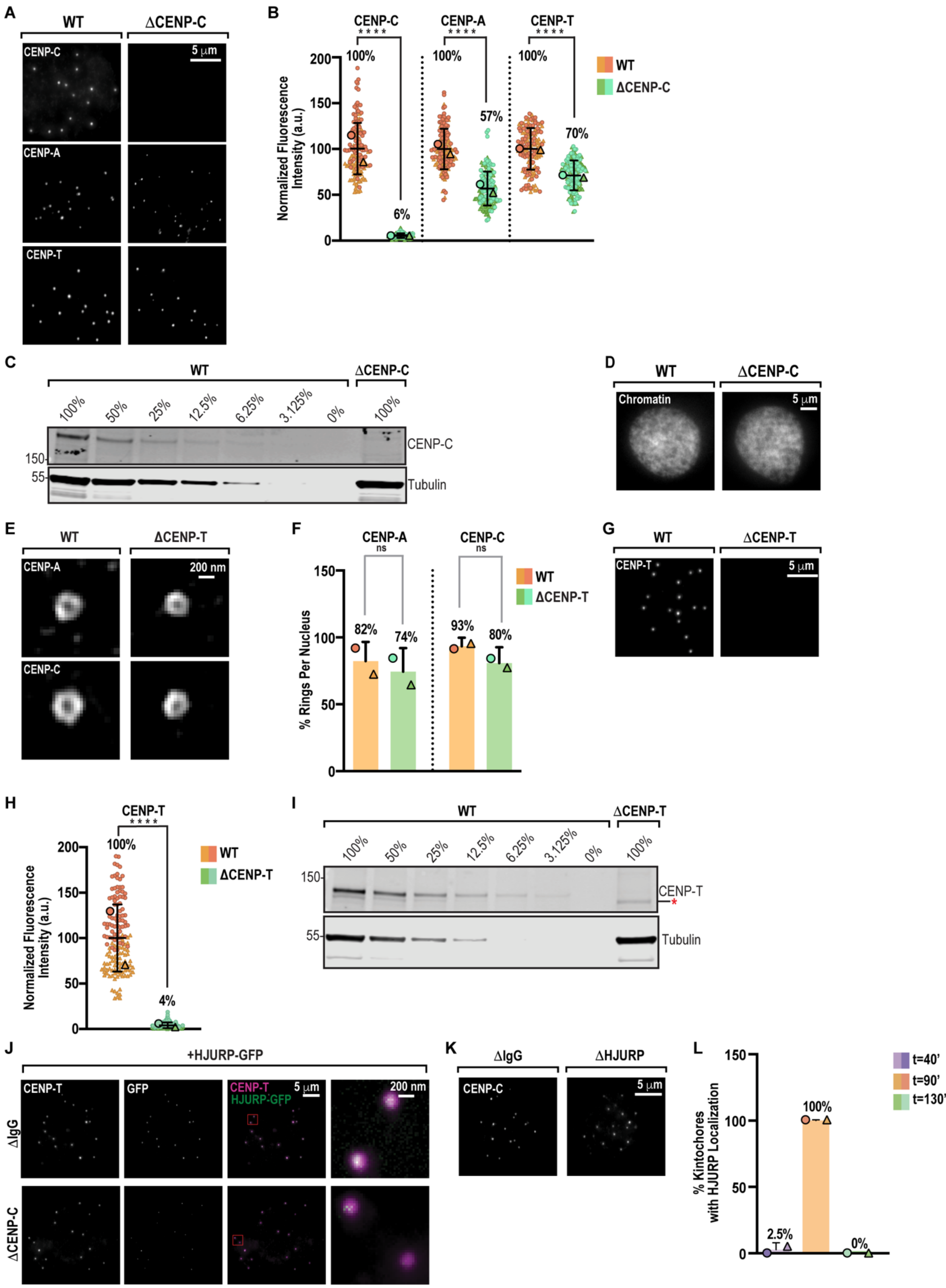

A
WT
ΔCENP-C
CENP-C
5 μm
CENP-A
CENP-T
B
CENP-C
CENP-A
CENP-T
****
100%
6%
57%
70%
WT
ΔCENP-C
Normalized Fluorescence Intensity (a.u.)
200
150
100
50
0
C
WT
ΔCENP-C
100%
50%
25%
12.5%
6.25%
3.125%
0%
CENP-C
Tubulin
150
55
D
WT
ΔCENP-C
Chromatin
5 μm
E
WT
ΔCENP-T
CENP-A
CENP-C
200 nm
F
CENP-A
CENP-C
ns
82%
74%
93%
80%
WT
ΔCENP-T
% Rings Per Nucleus
G
WT
ΔCENP-T
CENP-T
5 μm
H
CENP-T
****
100%
4%
WT
ΔCENP-T
Normalized Fluorescence Intensity (a.u.)
I
WT
ΔCENP-T
100%
50%
25%
12.5%
6.25%
3.125%
0%
150
55
CENP-T
*
Tubulin
J
+HJURP-GFP
CENP-T
GFP
CENP-T
HJURP-GFP
5 μm
200 nm
ΔIgG
ΔCENP-C
K
ΔIgG
ΔHJURP
CENP-C
5 μm
L
t=40'
t=90'
t=130'
2.5%
100%
0%
% Kintochores with HJURP Localization
150
100
50
0

**Fig. S7. CENP-C, but not CENP-T, is required for centromere architecture in interphase.** (**A**) Representative immunofluorescence of CENP-C, CENP-A, and CENP-T in interphase nuclei ($t$ = 90′), assembled from WT or ΔCENP-C extract, for Fig. 4F. Scale bar, 5 µm. (**B**) CENP-C, CENP-A, and CENP-T fluorescence intensities from (A), normalized to WT. SuperPlot of two biological replicates, replicate means color-coded. n = 75 centromeres or kinetochores per condition (≥ 6 nuclei). A.U., arbitrary units. Error bars, SD; ****, $P$ < 0.0001. (**C**) Western blot for CENP-C and tubulin in WT and ΔCENP-C extract from the samples in (A) and Fig. 4F. (**D**) Representative immunofluorescence of DNA (Hoechst) in interphase nuclei ($t$ = 90′) from ΔIgG and ΔCENP-C extract. Average cross-sectional nuclear area, ΔIgG: 393 +/- 73 $\mu m^2$, ΔCENP-C: 402 +/- 86 $\mu m^2$. n = 40 nuclei per condition. Scale bar, 5 µm. (**E**) Representative 3D-SIM images of CENP-A and CENP-C in interphase nuclei ($t$ = 90′), assembled from WT or CENP-T-depleted (ΔCENP-T) extract. Scale bar, 200 nm. (**F**) Percentage of observable CENP-A or CENP-C ring-like structures per nucleus for (E). SuperPlot of two biological replicates, replicate means color-coded. n = 8 nuclei per condition (≥ 129 centromeres). Error bars, SD; ****$P$ < 0.0001. (**G**) Representative immunofluorescence of CENP-T in interphase nuclei ($t$ = 90′) from WT or ΔCENP-T extract. Scale bar, 5 µm. (**H**) CENP-T fluorescence intensity from (G), normalized to WT. SuperPlot of two biological replicates, replicate means color-coded. n = 75 kinetochores per condition (≥ 6 nuclei). A.U., arbitrary units. Error bars, SD; ****, $P$ < 0.0001. (**I**) Western blot for CENP-T and tubulin in WT and ΔCENP-T extract from the samples in (E). The asterisk indicates a non-specific band. (**J**) Representative immunofluorescence of CENP-T (magenta) and HJURP-GFP (green) in interphase nuclei ($t$ = 90′), assembled from ΔIgG or ΔCENP-C extract containing *in vitro*-translated HJURP-GFP, for Fig. 4H. Scale bar, 5 µm. (**K**) Representative immunofluorescence of CENP-C in interphase nuclei ($t$ = 90′) from ΔIgG or ΔHJURP extract, for Fig. 4I. Scale bar, 5 µm. (**L**) Percentage of centromeres with HJURP-GFP present on the interphase nuclei and mitotic chromosomes in Fig. 4J. SuperPlot of two biological replicates, replicate means color-coded. n = 75 kinetochores per condition (≥ 5 nuclei).
